# Genome-wide identification, comprehensive characterization of transcription factors, cis-regulatory elements, protein homology, and protein interaction network of DREB gene family in *Solanum lycopersicum*

**DOI:** 10.3389/fpls.2022.1031679

**Published:** 2022-11-24

**Authors:** Hajra Maqsood, Faiza Munir, Rabia Amir, Alvina Gul

**Affiliations:** Department of Plant Biotechnology, Atta-ur-Rahman School of Applied Biosciences, National University of Sciences and Technology, Islamabad, Pakistan

**Keywords:** *Solanum lycopersicum*, DREB gene family, cis-regulatory elements, protein modeling, interactive association networks, transcriptome analysis

## Abstract

Tomato is a drought-sensitive crop which has high susceptibility to adverse climatic changes. Dehydration-responsive element-binding (DREB) are significant plant transcription factors that have a vital role in regulating plant abiotic stress tolerance by networking with DRE/CRT *cis*-regulatory elements in response to stresses. In this study, bioinformatics analysis was performed to conduct the genome-wide identification and characterization of DREB genes and promoter elements in *Solanum lycopersicum*. In genome-wide coverage, 58 *Sl*DREB genes were discovered on 12 chromosomes that justified the criteria of the presence of AP2 domain as conserved motifs. Intron–exon organization and motif analysis showed consistency with phylogenetic analysis and confirmed the absence of the A3 class, thus dividing the *Sl*DREB genes into five categories. Gene expansion was observed through tandem duplication and segmental duplication gene events in *Sl*DREB genes. Ka/Ks values were calculated in ortholog pairs that indicated divergence time and occurrence of purification selection during the evolutionary period. Synteny analysis demonstrated that 32 out of 58 and 47 out of 58 *Sl*DREB genes were orthologs to *Arabidopsis* and *Solanum tuberosum*, respectively. Subcellular localization predicted that *Sl*DREB genes were present in the nucleus and performed primary functions in DNA binding to regulate the transcriptional processes according to gene ontology. Cis-acting regulatory element analysis revealed the presence of 103 motifs in 2.5-kbp upstream promoter sequences of 58 *Sl*DREB genes. Five representative *Sl*DREB proteins were selected from the resultant DREB subgroups for 3D protein modeling through the Phyre2 server. All models confirmed about 90% residues in the favorable region through Ramachandran plot analysis. Moreover, active catalytic sites and occurrence in disorder regions indicated the structural and functional flexibility of *Sl*DREB proteins. Protein association networks through STRING software suggested the potential interactors that belong to different gene families and are involved in regulating similar functional and biological processes. Transcriptome data analysis has revealed that the *Sl*DREB gene family is engaged in defense response against drought and heat stress conditions in tomato. Overall, this comprehensive research reveals the identification and characterization of *Sl*DREB genes that provide potential knowledge for improving abiotic stress tolerance in tomato.

## Introduction

Tomato (*Solanum lycopersicum* L.) is an economically valuable fruit worldwide. Its yield and development are acutely affected by harsh environmental conditions such as drought, salinity, and extreme temperatures. Salinity and drought stress conditions lead to harmful physiological effects—for instance, stunted plant growth, failure in seed germination, and reduction in fruit yield. Moreover, drought causes osmotic stress that results in the decrease of photosynthetic rate and plant development (Diouf et al., 2018; Zhou et al., 2019). In the FAO 2019 report, the data stated a substantial increase in tomato production to 182 million tons from 37 million tons that was in 2017.

The dehydration-responsive element-binding (DREB) gene family is one of the most prominent transcription regulators that bind with DRE/cis regulatory elements (CREs) to induce tolerance under biotic and abiotic stresses in plants (Du et al., 2018). It is also known as cis-binding factor, belongs to the ethylene-responsive element-binding factor (ERF) family, and contains conserved AP2 domain (Khan, 2011). The presence of valine (V) and glutamic acid (E) at the 14th and 19th positions in the AP2 domain adds more clarity to the DREB family from its other sub-family members. The AP2 domain contains 60 to 70 amino acid sequences that are responsible for plant defense mechanisms to combat stressful circumstances (Wu et al., 2015). YRG (19–22 aa long) and RAYDS are two conserved elements that are characteristic features of the AP2 domain. The core region of RAYD is supposed to be involved in the existence of an amphipathic *α*-helix (Chai et al., 2020). DREB is a sub-family of ERF transcription factors with conserved AP2 domain. The three-dimensional structure of AP2 domain was revealed in *At*ERF1 gene through nuclear magnetic resonance imaging that demonstrated the presence of an α-helix and three antiparallel β-sheets in its structural organization (Lata and Prasad, 2011; Li Z. et al., 2021). DREB genes attach to drought-responsive (DRE/CRT) elements with the assistance of a V residue, two W residues, and four R residues (Mushtaq et al., 2021). The phosphorylation of DREB genes is essential for their activation, supported by a threonine- or serine-rich sequence present next to the AP2 domain (Lata and Prasad, 2011). The DRE/CRT elements have a core motif consisting of ACCGAC/GCCGAC that directly interacts with DREB genes under abiotic stresses (Vazquez-Hernandez et al., 2017). The DREB gene family further splits into six classes, *i*.*e*. A1–A6, based on their fundamental structural and functional characteristics (Chai et al., 2020).

Subsequently, the DREB gene family has been identified in a number of plants including *Arabidopsis* (Hwang et al., 2012), pearl millet, strawberry, tobacco (Cheng et al., 2013), rice, lettuce (Park et al., 2020), bananas, wheat, bell pepper, maize (Liu et al., 2013), soybean (Hou et al., 2022), and potato (Mushtaq et al., 2021). Previous studies have identified DREB1, DREB2, and DREB3 in tomatoes. *Sl*DREB1 has a remarkable role in response to drought stress. It enhances the concentration of soluble sugars and accumulates osmolytes that further balance the osmotic pressure and increase the tolerance response under water-deficient conditions (Jiang et al., 2017). *Sl*DREB2 is involved in the regulation of stress signaling pathways. Studies have shown its contribution in the development of early vegetative and reproductive stages. It regulates the synthesis of proline that further assists plants in response to salt and hormonal changes (Hichri et al., 2016), whereas *Sl*DREB3 is a negative regulator of the abscisic acid (ABA) pathway by altering ABA signaling. It is also known as the ripening gene by controlling the ethylene-dependent and ethylene-independent pathways (Gupta et al., 2022).

Despite this, limited studies have been conducted on the characterization of the DREB gene family in *S. lycopersicum*. The present research data extensively covers phylogenetic evaluation, gene structure analysis, conserved motifs, chromosomal localization, gene duplication events, synteny analysis for the detection of orthologues in interspecies, gene ontology, characterization of cis-regulatory elements, prediction of subcellular localization, protein modeling, prediction of pocket binding sites, disorder region analysis, and interactive networks with other proteins. Overall, our data has the potential information that serves as a strong infrastructure for unravelling the extensive structural and functional relationships of intricate genes for genetic engineering to enhance tolerance in tomato genotypes under environmental stresses.

## Materials and methods

### Identification and sequence conservation analysis of DREB family members in *Solanum lycopersicum*

To retrieve the amino acid sequences of DREB family members, the amino acid sequences of DREB genes of *Arabidopsis thaliana* were retrieved from the TAIR database (https://www.arabidopsis.org/) (Huala et al., 2001). These amino acid sequences were used as query sequences in BLASTp for the retrieval and identification of DREB family members in *Solanum lycopersicum* from Sol Genomics Networks (SGN) (https://solgenomics.net/). The criteria for BLASTp homology search was set to 1 × 10^-5^ against *Solanum* lycopersicum genome v4.0 (Mueller et al., 2005).

ScanProsite Server (https://prosite.expasy.org/scanprosite/) was used to identify a single AP2 domain, its sequence, and amino acid length throughout the DREB family (De Castro et al., 2006) NCBI CDD (https://www.ncbi.nlm.nih.gov/Structure/cdd/cdd.shtml) (Yang et al., 2020) and Pfam (http://pfam.xfam.org/) (Mistry et al., 2021) were used for the verification of the complete AP2 domain. The ExPASY protparam tool (https://web.expasy.org/protparam/) (Gasteiger et al., 2005) was used to measure physicochemical properties such as the molecular weight (kDa), protein length, and isoelectric point (pI) of DREB protein sequences.

Verification of the conserved amino acid valine (V) at the 14th position and glutamic acid (E) at the 19th position alignment was conducted throughout the DREB protein sequences in *S. lycopersicum* which were retrieved through BLASTp using MEGA 11 software (www.megasoftware.net) using default settings (Tamura et al., 2021).

### Phylogenetic analysis

To classify *Sl*DREB family members, retrieved amino acid sequences of Arabidopsis and *S. lycopersicum* were used. MEGA11 (www.megasoftware.net) software was used with default parameters for the multiple sequence alignment. Criteria for the construction of phylogenetic trees on MEGA 11 software (www.megasoftware.net) was set to maximum likelihood method with pairwise deletion, using the Poisson substitution model with bootstrap set to 1000 replicates (Tamura et al., 2021). The phylogenetic tree was used for the DREB family members classification in *S. lycopersicum* based on the classes in *At*DREB proteins.

### Intron–exon organization, motif analysis, and genomic localization

To generate the gene structure graph, Gene Structure Display Server (GSDS 2.0) (http://gsds.gao-lab.org0) was used which shows the number of introns and exons within a sequence (Hu et al., 2015). For this purpose, genomic and coding sequences (CDs) of all *Sl*DREB genes were retrieved from the Phytozome. Comparison between these two sequences assist to generate intron-exon graphical illustration.

To detect motifs within the classes of *Sl*DREB family members, MEME software version 5.3.2 (https://memesuite.org/meme/tools/meme) was selected and set parameters such as (i) an optimal width of motifs was 6-50 amino acids, (ii) zero or one occurrence per sequence of a single motif within the model, and (iii) number of motifs set to 20 (Bailey et al., 2015).

### Genomic localization, gene duplication events, and synteny analysis

To map chromosomal positions of *Sl*DREB genes, Toolkit Biologists Tools (TBtools) (https://github.com/CJ-Chen/TBtools) (Chen et al., 2018) software was used for the illustration of distances and physical location of *Sl*DREBs.To evaluate respective time of divergence and selective pressure, Toolkit Biologists Tools (TBtools) software (https://github.com/CJ-Chen/TBtools) was used to measure the Ka and Ks values that denote non-synonymous and synonymous values. To calculate divergence time following formula was used T=Ks/2x*MYA, where x=6.56 x 10^-9^ and MYA= 10^-6^ (Yuan et al., 2015). Gene expansion follows two methods in gene duplication events. First method is tandem repeats if two genes present on the same chromosome are separated by fewer loci. Second method of gene expansion is segmental duplication (Laha et al., 2020).

For orthologues and synteny analysis of *Sl*DREB genes with *A. thaliana* and *S. tuberosum*, gff3 annotation and genomic files were downloaded from Phytozome 13v database. To access the segmental duplication gene pairs Circos plot was constructed through Advanced Circos software and Dual synteny plotter was used for the visualization of orthologues of *S. lycopersicum* in the *A. thaliana* and *S. tuberosum* using TB Tools (https://github.com/CJ-Chen/TBtools) respectively.

### Gene ontology annotation, functional analysis, and subcellular localization prediction

To interpret Gene Ontology (GO), transcript sequences of *Sl*DREBs were uploaded to Blast2Go software (https://www.blast2go.com/) (Conesa and Götz, 2008). Functions like functional annotation, BLASTx search, InterProScan, mapping, and annotation were executed with default parameters. Furthermore, these sequences were subjected to the investigation and graphical representation of biological processes (BP), cellular compartments, and molecular functions. The WoLF PSORT tool (https://wolfpsort.hgc.jp/) was used for the estimation of the subcellular localization of DREB genes (Horton et al., 2007).

### Cis-regulatory element analysis of *Sl*DREB gene promoters

To examine the promoter regions, Phytozomev13 database (https://phytozome.jgi.doe.gov/pz/portal.html) (Goodstein et al., 2012) was used for the retrieval of a 2.5-kbp upstream promoter sequence of the *Sl*DREB genes of *S. lycopersicum* v4.0 genome. The promoter is an upstream region from the start site (ATG) which encodes for methionine (M) that signals for the translational start site. SGN (https://solgenomics.net/) was used to validate these promoter sequences. Plant CARE database (http://bioinformatics.psb.ugent.be/webtools/plantcare/html/) (Lescot et al., 2002) was used for the estimation of CREs. Toolkit Biologists Tools (TBtools) software was used to illustrate the graphical positions of CREs in sequences.

### Secondary and three-dimensional protein modeling

Five members were selected from five subgroups identified from the present study. The amino acid sequences of these members were used to perform secondary structure evaluation using the SOPMA server (https://npsaprabi.ibcp.fr/NPSA/npsa_sopma.html) (Geourjon and Deleage, 1995). To predict the 3D structure, the Phyre2 server (http://www.sbg.bio.ic.ac.uk/~phyre2/html/page.cgi?id=index) was employed on selected amino acid sequences. This software used modern methods for homology detection and assisted in the construction of reliable 3D structures (Kelley et al., 2015).

### Structural confirmation, prediction of catalytic sites, and disorder region analysis of *Sl*DREB proteins

The predicted models were saved in PDB format. These models were subjected to Galaxy Refine server (http://galaxy.seoklab.org/cgi-bin/submit.cgi?type=REFINE) (Heo et al., 2013), PROCHECK server, and Ramachandran plot analysis (https://servicesn.mbi.ucla.edu/PROCHECK/) (Laskowski et al., 2006) that assist in model refinement and structural validation. To visualize the refined stable structures of *Sl*DREB proteins, pdb-format files were utilized in ChimeraX (http://www.rbvi.ucsf.edu/chimerax/) software (Pettersen et al., 2021). To estimate the active catalytic sites, CASTp 3.0 (http://sts.bioe.uic.edu/) web server was used, whichs point out multiscale pockets on the structures of selected *Sl*DREBs members (Tian et al., 2018). PrDOS (Protein DisOrder prediction System) server (http://prdos.hgc.jp/cgi-bin/top.cgi) (Ishida and Kinoshita, 2007) was utilized for the prediction of disorder regions within protein with default settings.

### Interactive network analysis of *Sl*DREB proteins

Proteins interact with many other proteins either belonging to the same class or different groups in response to regulating different signaling pathways. To evaluate the interaction of *Sl*DREB proteins with other interactors, protein sequences of the selected *Sl*DREB proteins were utilized in STRING Tool (Search Tool for the Retrieval of Interacting Genes; https://string-db.org/) (Szklarczyk et al., 2019) for the construction of interactive networks. The criterion for the confidence threshold was set to 0.40, and a maximum number of 10 interactors were selected in the first shell. Interactive networks showed the interconnection of query protein with other 10 interactors; some of them are interconnected.

### Transcriptome analysis of tomato under drought and heat stress

The tomato transcriptome was retrieved from the Bio project NCBI (PRJNA635375) (https://www.ncbi.nlm.nih.gov/bioproject/635375). It encompasses gene expression data under drought and heat stresses through the Gene Expression Omnibus (GEO) NCBI (GSE151277) (https://www.ncbi.nlm.nih.gov/geo/) (Barrett et al., 2005). GEO datasets were refined through TBtools (https://github.com/CJ-Chen/TBtools) by using a table file manipulator to extract the gene expression of 58 *Sl*DREB genes present in the tomato transcriptome. Subsequently, log_2_ base values were calculated for the normalization of the original dataset. An expression heat map was constructed through TBtools heat map illustrator to illustrate the upregulation and downregulation of *Sl*DREB genes under drought and heat stress conditions.

## Results

### Identification of putative DREB family members in S. *lycopersicum*

Our results revealed 58 DREB protein sequences from tomato genome that show the maximum percentage identity and coverage with query sequences. The retrieved sequences, which were presented as *Sl*DREB1 to *Sl*DREB58, belong to DREB and ERF family according to Phytozome annotations (**Supplementary Table S1**). Scan prosite Server and CDD NCBI confirmed the occurrence of one conserved AP2 domain within each of the 58 *Sl*DREB sequences. The chromosomal location, conserved domain length, protein length, and physiological characteristics such as isoelectric point and molecular weight (Mw) are indicated in **Supplementary Table S1**. The protein sequences of 58 *Sl*DREB proteins are presented in **Supplementary Table S2**.

### Multiple sequence alignment and phylogenetic analysis

**Figure 1:**
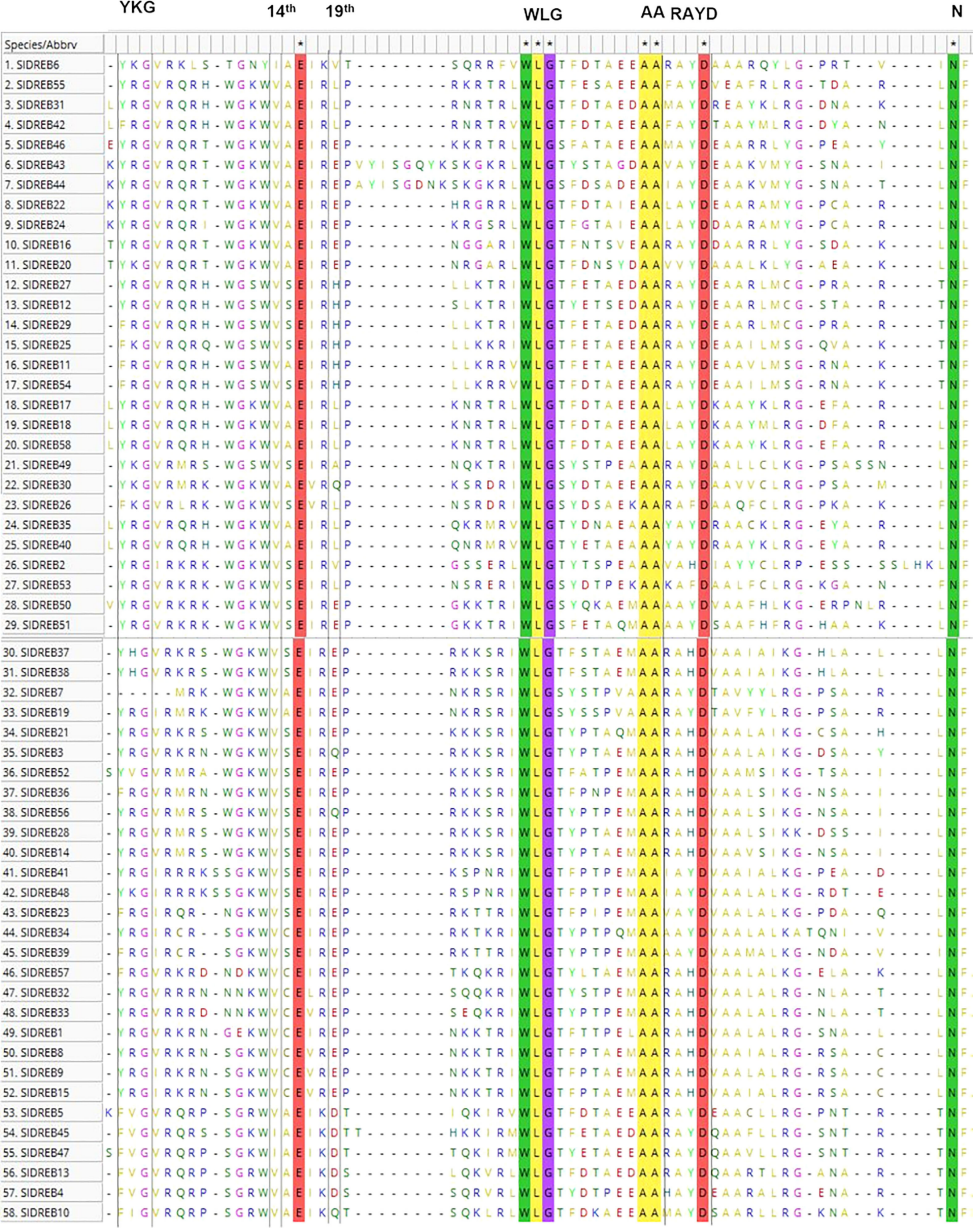
Multiple sequence alignment of *S. lycopersicum* dehydration-responsive element-binding (DREB) proteins. MEGA 11 software was employed using MUSCLE algorithm. The highlighted lines represent conserved amino acids and motifs, whereas motifs and amino acids enclosed in black lines represent variability and have active roles in protein structure and functionality.

Multiple sequence alignment proved that the amino acids valine (V) and glutamic acid (E) at positions 14th and 19th within the AP2 domain were not conserved in the case of *Sl*DREB sequences as demonstrated in **Figure 1**. Three *Sl*DREB protein sequences (*Sl*DREB6, *Sl*DREB45, and *Sl*DREB47) contain isoleucine (I) instead of valine (V) at position 14th. I can only replace V due to the same characteristics such as hydrophobicity, aliphatic, and nonpolar in physical as well as chemical features such as polarity. Among the 58 *Sl*DREB sequences, 36 *Sl*DREBs have (V) and (E) at the 14th and 19th positions. Glutamic acid (E) at 19th position is replaced by different amino acids like leucine (L) in seven *Sl*DREBs, histidine (H) in six *Sl*DREBs, valine (V) in two *Sl*DREBs, glutamine in four *Sl*DREBs, alanine (A) in one *Sl*DREB, and aspartic acid (D) in two *Sl*DREB sequences, respectively.

Among these amino acids, E and D are hydrophilic, positively charged, and polar, Q is hydrophilic, uncharged, and polar, H is hydrophilic, positively charged, heterocycle, and polar, L and V are hydrophobic and non-polar, and A is hydrophobic, neutral, and non-polar in nature correspondingly (**Supplementary Table S1**). These sequences have a conserved WLG motif in its AP2 domain. All these amino acid modifications and features have a prominent effect on the binding capacity and interactions of *Sl*DREB genes with DRE/GCC boxes and with other proteins in signaling pathways.

**Figure 2:**
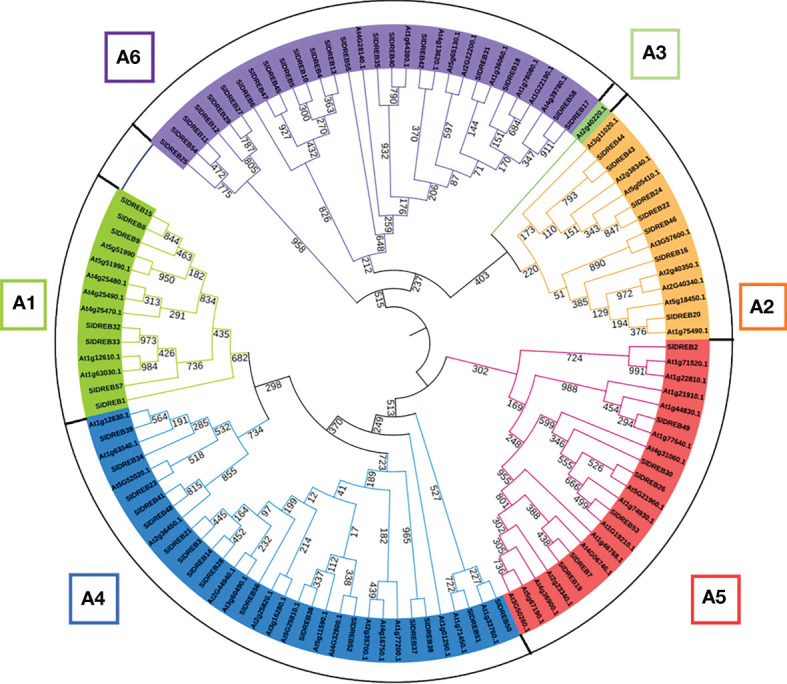
Phylogenetic tree of dehydration-responsive element-binding proteins of *S. lycopersicum* and *A. thaliana*. MEGA 11 software was utilized with 1,000 bootstrap and maximum likelihood method. Each color represents a specific class.

To identify DREB gene sub-classes, a phylogenetic analysis was assessed through MEGA 11 using MUSCLE algorithm at 1,000 bootstraps through the maximum likelihood method. Based on the classification in *A. thaliana* DREB family, the phylogenetic tree proved the absence of A3 sub-class in SlDREB genes because no *Sl*DREB gene showed a homology with AT2G40220.1 (green color) which belongs to A3 sub-class as in **Figure 2**. *Sl*DREB genes that show greater homology with each other are structurally more closely related than the *At*DREB genes. A further investigation of the motifs and gene structures of the 58 *Sl*DREB genes will prove their high homology.

### Intron–exon organization and motif analysis

**Figure 3:**
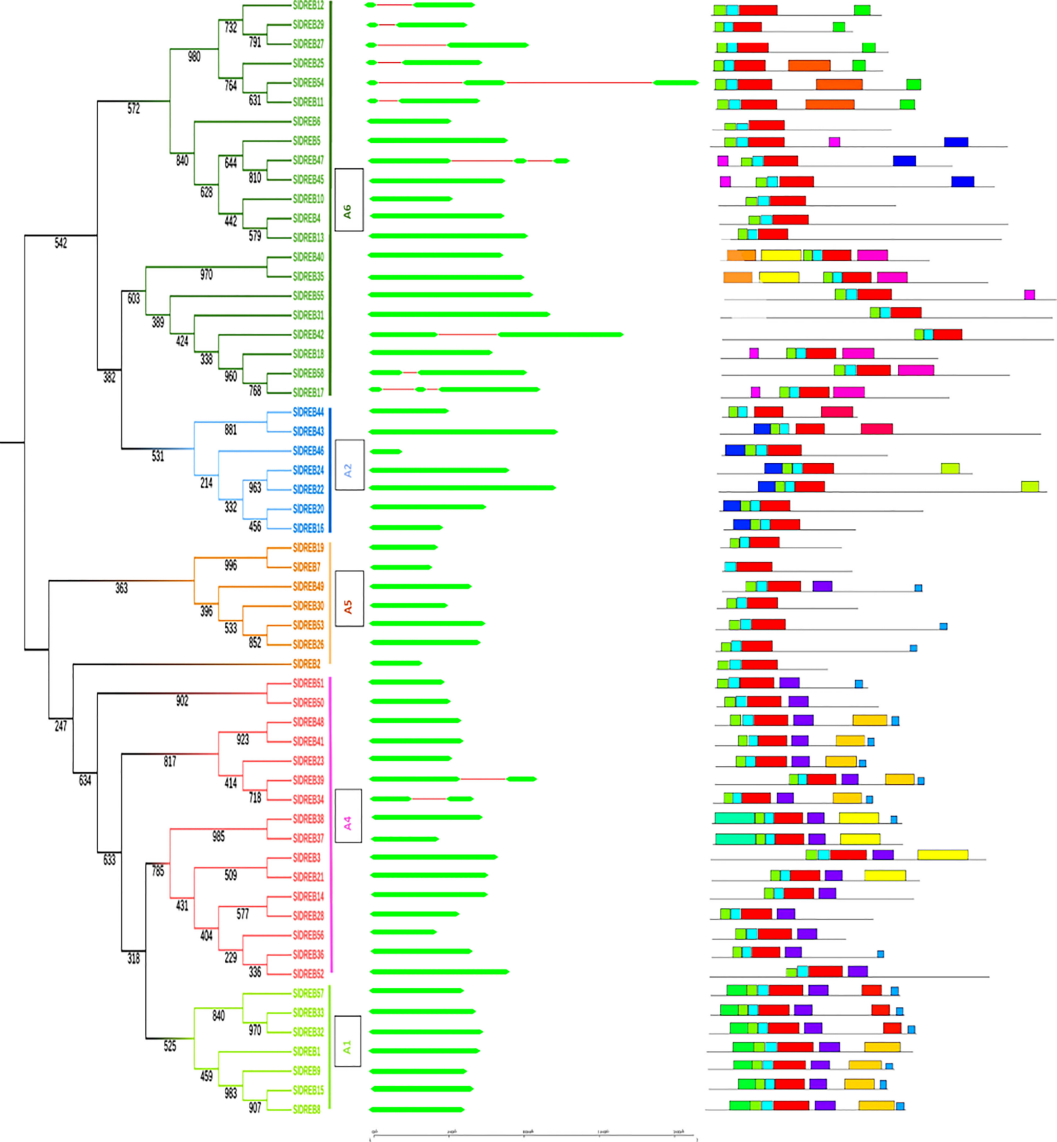
Intron–exon organizations of *Sl*DREB genes. Gene structure was analyzed using Gene Structure Display Server. Here exons and introns are represented with green and red colors, respectively, whereas the scale shows the corresponding sizes of the genes. Twenty motifs predicted through MEME software within SlDREB protein sequences. The motifs were differentiated by distinctly colored boxes, whereas gray lines represented non-conserved regions.

Gene structure is very crucial in the analysis of structural diversity of duplicated genes. Intron and exon organization reflects intuition about the structural diversity and similarity of DREB genes belonging to different classes in tomato. For the graphical illustration of gene structure organization, full-length cDNA was aligned and compared with genomic DNA of *Sl*DREB sequences (**Supplementary Table S2**). The results indicated that only one intron and two exons were present in nine *Sl*DREB genes (11, 12, 25, 27, 29, 34, 39, 42, and 58) and two introns and three exons were observed in the coding region of three *Sl*DREB genes (17, 47, and 54). Except these 12 *Sl*DREB genes, 46 other genes had only one exon in its coding region subsequent to the phylogenetic tree (**Figure 3**). A detailed motif analysis was conducted through MEME software as summarized in **Supplementary Table S3**). It assisted in the clarity of diversity among 58 *Sl*DREB genes belonging to different classes. The results showed the variability in motifs of one class from another (**Supplementary Table S4**). A total of 20 motifs were discovered, from which motifs 1, 2, and 3 evaluated the presence of the AP2 domain which is a characteristic domain of DREB proteins. Motifs 1, 2, and 3 were present in all S*l*DREB proteins, with some degree of divergence except *Sl*DREB7 (which belongs to A5 class) which showed the absence of motif 3 only. A1 class had motif 6 in all of its members and motif 17 in *Sl*DREBs 32, 33, and 57. In A2 class, motif 7 was present in all of its members except *Sl*DREB43, with motif 16 in *Sl*DREBs 43 and 44 and motif 20 in *Sl*DREBs 22 and 23. A4 class had motifs 10 and 12 in its members as follows: *Sl*DREB 3, 21, 37, and 38 and *Sl*DREB 37 and 38, respectively. A5 class shared only a few motifs with A1 and A4 classes. The following motifs corresponding to *Sl*DREB genes were identified specifically in A6 class: motif 8 in 17, 18, 35, 40, and 58; motif 9 in 11, 25, and 54; motif 11 in 11, 12, 27, 25, 29, and 54; motif 14 in 5, 45, and 47; motif 15 in 5, 17, 18, 45, 47, and 55; and motifs 18 and 19 in 35 and 40 *Sl*DREB genes, respectively. Motif 4 was observed in the entire A1 and A4 classes and in a member of A5 class (*Sl*DREB49). Motif 5 was detected in a few members of A1 and A4 classes. Motif 13 was identified in A1, A4, and A5 groups. The same motif structure was observed among members of the same class. A motif analysis compared with a phylogenetic tree proved the evolutionary similarity of A1, A4, and A5 sub-classes that appeared in the same clade and their diversity with A2 and A6 classes which were associated in the second clade as presented in **Figure 3**.

### Genomic localization, gene duplication events, and synteny analysis

**Figure 4:**
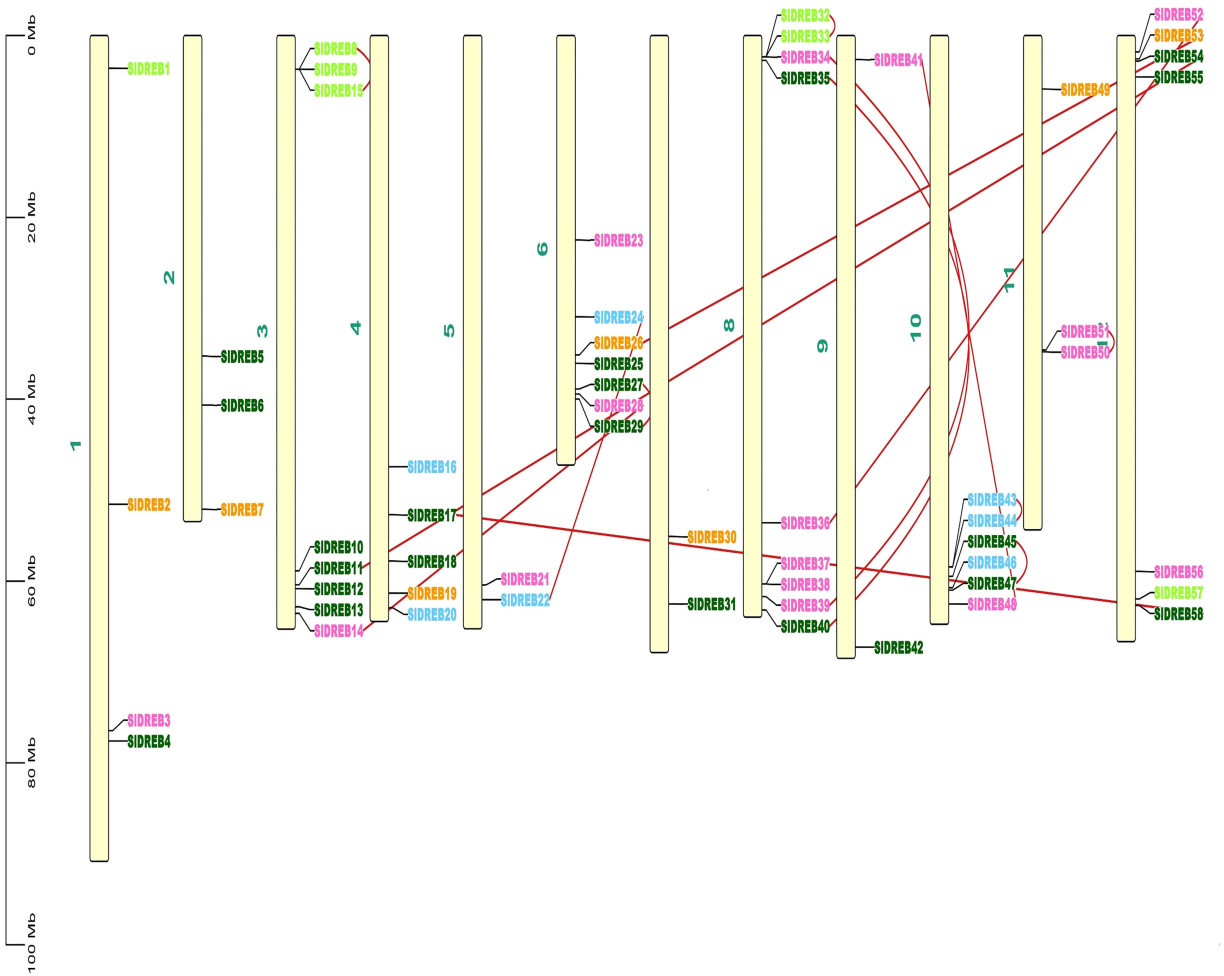
Chromosomal distribution of 58 SlDREB genes within the genome was illustrated by using TBtools. Each color of the SlDREB genes represents its class according to the phylogenetic tree. The scale shows the length of each chromosome. Red lines that linked the gene pairs present on the same chromosome denoted tandem duplication events.

**Figure 5:**
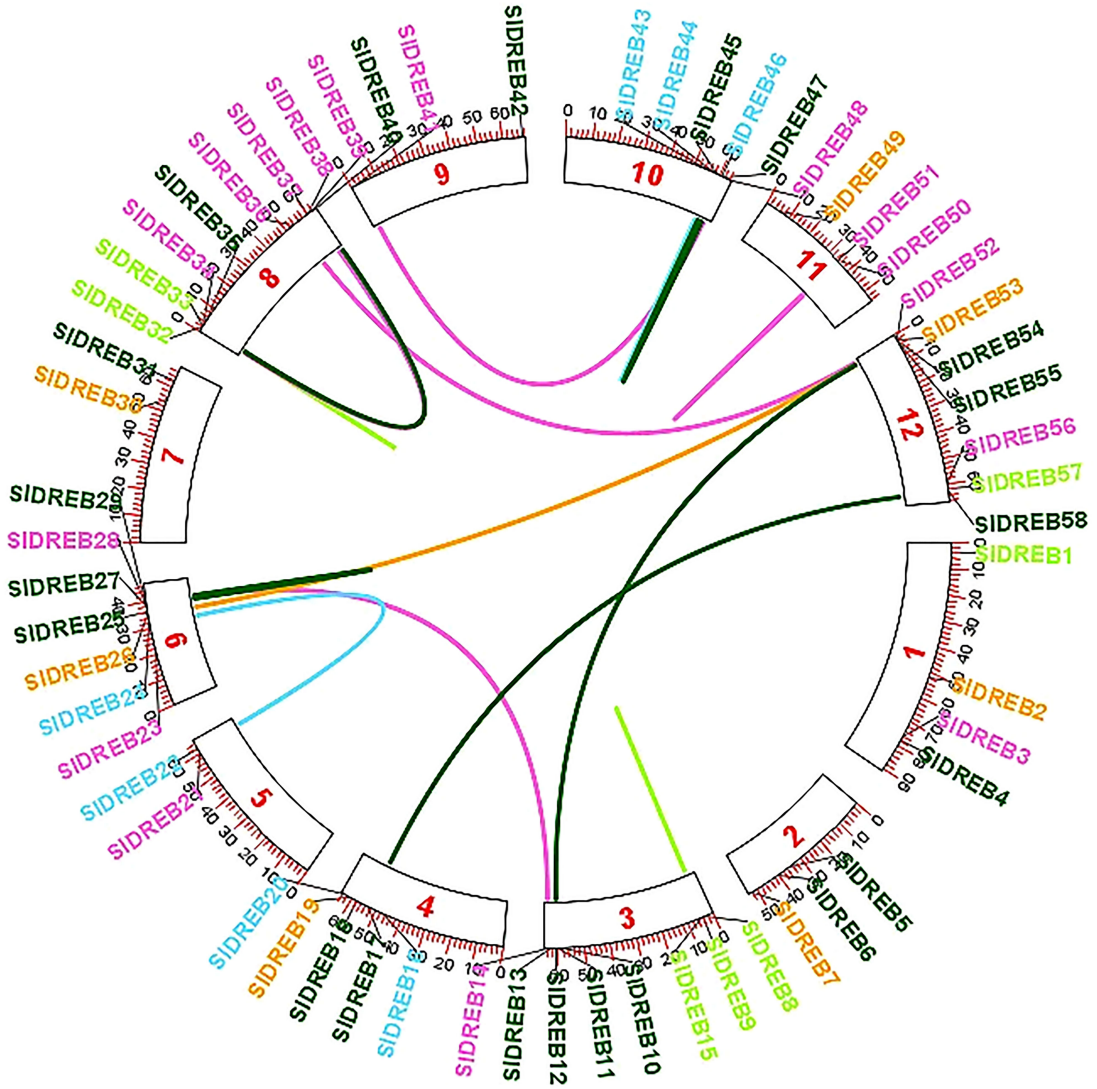
Segmental gene duplications were exhibited on the Circos plot of 58 SlDREB genes that was generated by the respective positions of each gene across 12 chromosomes. Colored lines indicated the segmental gene duplications among gene pairs situated on different chromosomes.

To localize the 58 *Sl*DREB genes, chromosomal localization was retrieved from Phytozome v.13 with its full length. TB Tools software was utilized for the physical mapping and distribution of all *Sl*DREB genes across the tomato genome. All 58 *Sl*DREB genes were represented in different colors that denoted their respective classes as indicated in the rectangular phylogenetic tree. Mapping demonstrated that genetic variation was observed in the gene distribution on 12 chromosomes. Chromosome 8 (chr08) had nine *Sl*DREB genes positioned on it, which is the largest number compared with other chromosomes. However, three chromosomes (chr05, chr07, and chr09) comprised the least number of *Sl*DREB genes which is 2. Chromosome 1 (chr01) had four *Sl*DREB genes, chromosomes 2 and 11 (chr02 and 11) had three *Sl*DREB genes, chromosome 3 (chr03) was comprised of eight *Sl*DREB genes, chromosome 4 (chr04) consisted of five *Sl*DREB genes, chromosomes 6 and 12 (chr06 and chr12) covered seven *Sl*DREB genes, and chromosome 10 (chr10) positioned six *Sl*DREB genes, respectively, as depicted in **Figure 4**. The plant genome undergoes genomic evolution due to gene expansion events that introduce new interactions within a genome and assist in the sustainability of unique functions. Two types of duplication events were observed in the tomato genome: tandem and segmental duplication. Tandem duplications were observed in eight gene pairs in which chromosome 8 (chr08) covered a large number of gene pairs which is three, namely, *Sl*DREB 32–33, 34–39, and 35–40. Conversely, chromosomes 3, 6, and 11 (chr03, chr06, and chr11) was each comprised of only one gene pair (8–15), (27–29), and (50–51). Two pairs were positioned on chromosome 10 (chr10), which were *Sl*DREB 43–44 and 45–47, represented by the red lines as given in **Figure 4**. However, segmental gene duplication event was observed in seven *Sl*DREB gene pairs (36–52, 26–53, 11–54, 41–48, 17–58, 14–28, and 22–24) positioned on different chromosomes linked though the red lines in the Circos plot as illustrated in **Figure 5**.

**Figure 6:**
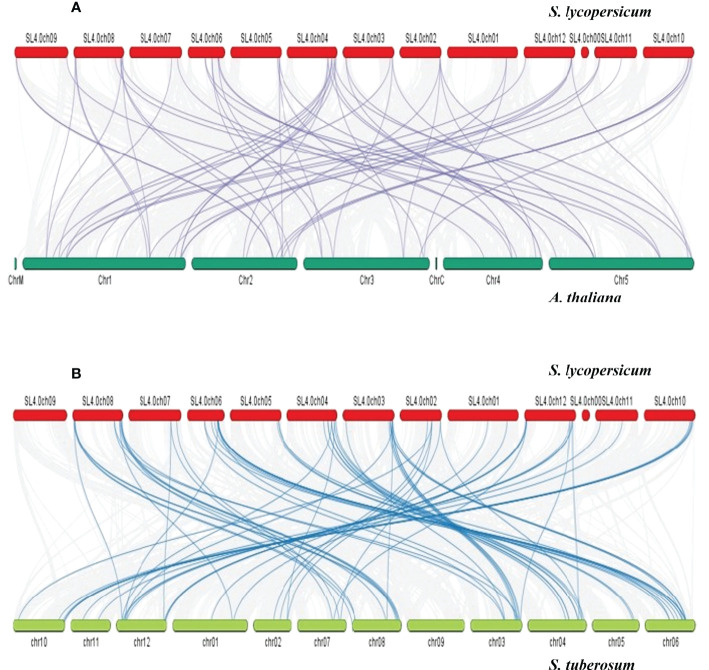
Synteny analysis was conducted among **(A)** *S. lycopersicum* and *A thaliana* and **(B)** *S. lycopersicum* and *S. tuberosum* for homology and evolutionary relationship. The red, dark green, and green bars denote the chromosomes of *S. lycopersicum*, *A thaliana*, and *S. tuberosum*. The gray lines indicate the collinear block within genomes. The purple and dark blue lines were designated to the respective homolog dehydration-responsive element-binding genes present between genomes.

The Ka/Ks values of these 15 *Sl*DREB gene pairs were measured for the estimation of divergence time and type of selection in each pair. If the Ka/Ks value is equal to 1, then the gene pair shows neutral selection. Although purification selection occurred in a gene pair if the Ka/Ks value was less than 1, on the contrary, Ka/Ks values less than 1 were denoted as a positive selection. In 15 pairs of *Sl*DREB genes, duplication events occurred through purification selection and had a divergence time between 4.0 and 50 million years ago as represented in **Supplementary Table S5**. Synteny analysis was conducted for the evolutionary relationship that exhibited the collinearity of *Sl*DREB genes in *A. thaliana* and in *S. tuberosum* by comparing their genomes with *S. lycopersicum* (**Figure 6**). The syntenic map revealed that 32 out of 58 *Sl*DREB genes displayed a high-homology relationship in *A. thaliana*, whereas 47 out of 58 *Sl*DREB genes revealed their orthologs in *S. tuberosum* as shown in **Supplementary Table S5**.

### Gene ontology and subcellular localization

**Figure 7:**
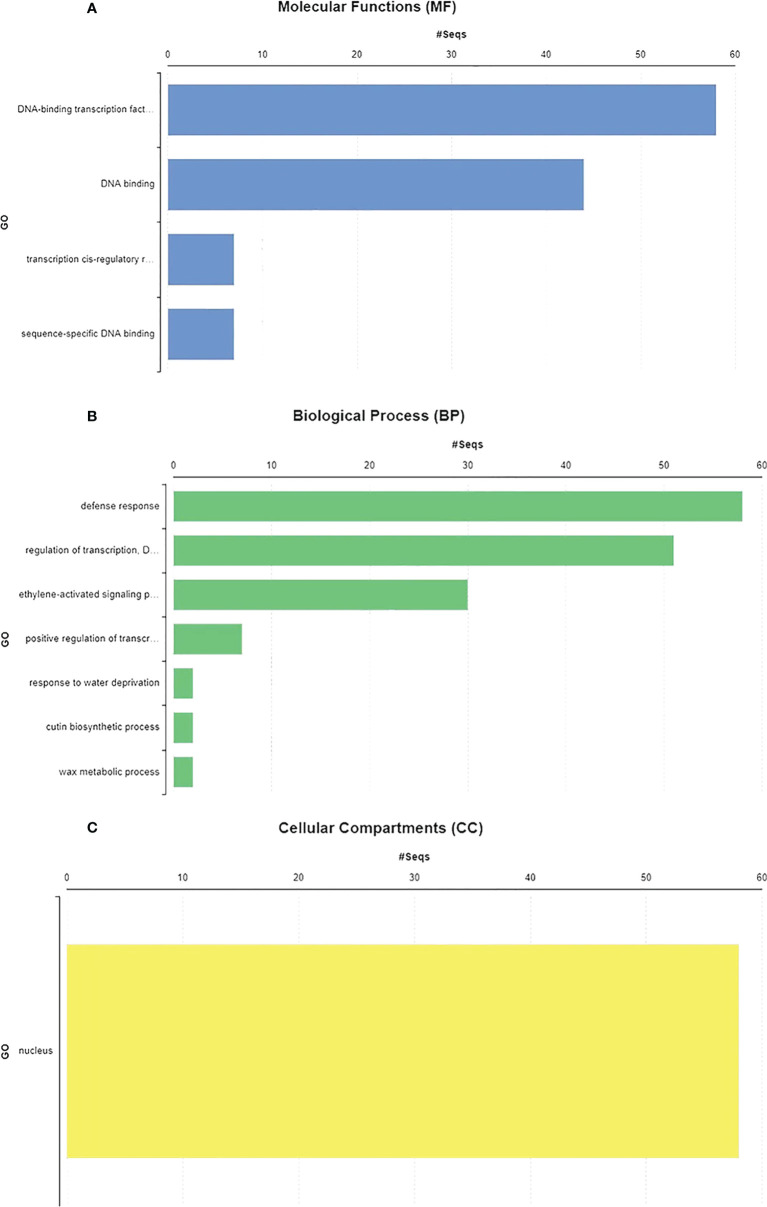
Gene ontology and functional analysis were performed by using Blast2GO software. **(A)** SlDREB genes involved in the respective molecular functions. **(B)** Biological processes conducted by these genes. **(C)** Cellular compartments comprised by SlDREB genes.

**Figure 8:**
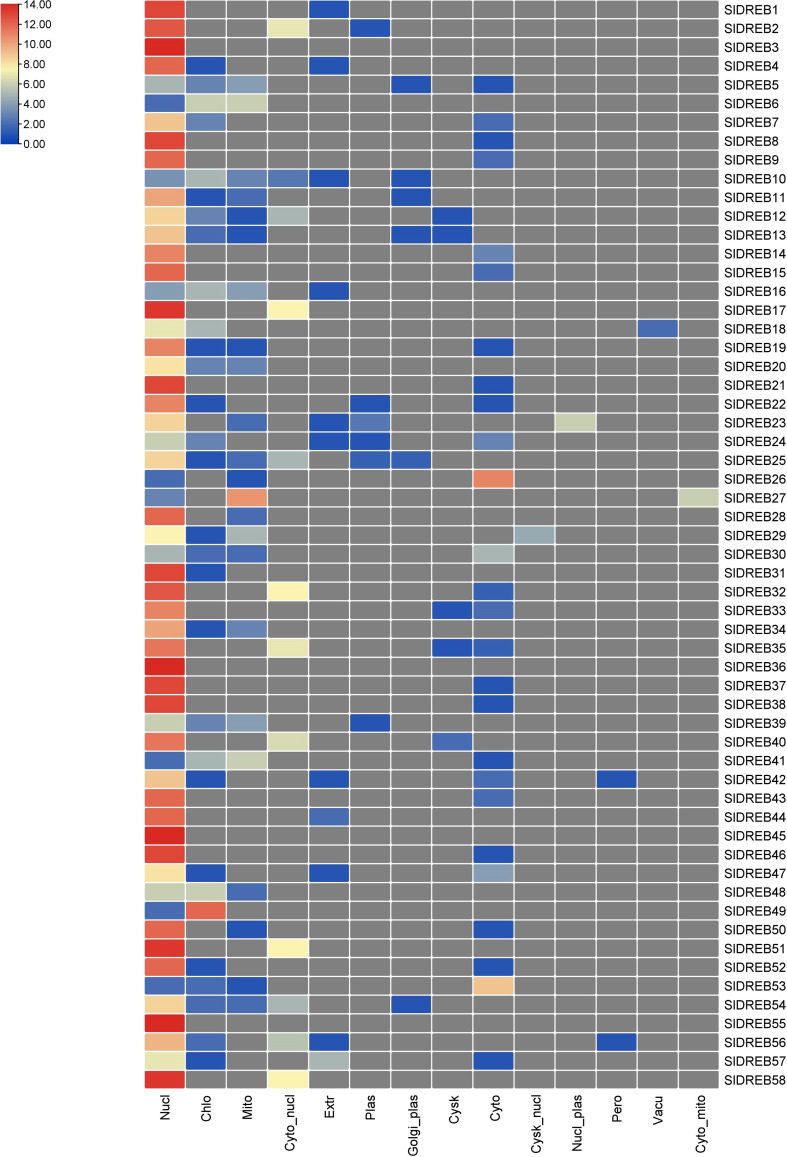
Illustration of cellular localization prediction of 58 SlDREB proteins. Each color indicates the level of abundance of SlDREB proteins in different cellular compartments.

GO annotations and Eggnog descriptions were performed on 58 *Sl*DREB genes, followed by gene mapping, InterPro scan, and blast hits. Molecular functions, biological functions, and localization in cellular compartments of *Sl*DREB genes were estimated. The results revealed, as shown in **Figure 7A**, that *Sl*DREB genes were specifically involved in the transcriptional regulations, sequence-specific DNA binding that proved the involvement of these genes in stress-responsive gene regulations, growth, and development of tomato. Under molecular function analysis, *Sl*DREB genes were involved in the following processes: 58 *Sl*DREB genes play a role in defense mechanisms, 51 *Sl*DREB genes regulate transcription factors, seven genes (*Sl*DREB 16, 20, 22, 24, 43, 44, and 46) were involved in positive transcriptional regulation, two *Sl*DREBs (11 and 54) respond to water deprivation, two *Sl*DREBs (11 and 54) were linked with cutin biosynthesis, and two genes (*Sl*DREB 11 and 54) were involved in the metabolic process as given in **Figure 7B**. The GO analysis showed that all 58 *Sl*DREB genes were localized in the nucleus, as shown in **Figure 7C** and supported by **Supplementary Table S6**. The further subcellular localization of *Sl*DREB genes was confirmed by WoLF PSORT software, and the results were depicted in the form of a heat map which exposed that *Sl*DREB genes were highly concentrated in the nucleus following 13 other organelles, as shown in **Figure 8** (**Supplementary Table S6**).

### Cis regulatory element analysis in the promoter regions of 58 *Sl*DREBs

**Figure 9:**
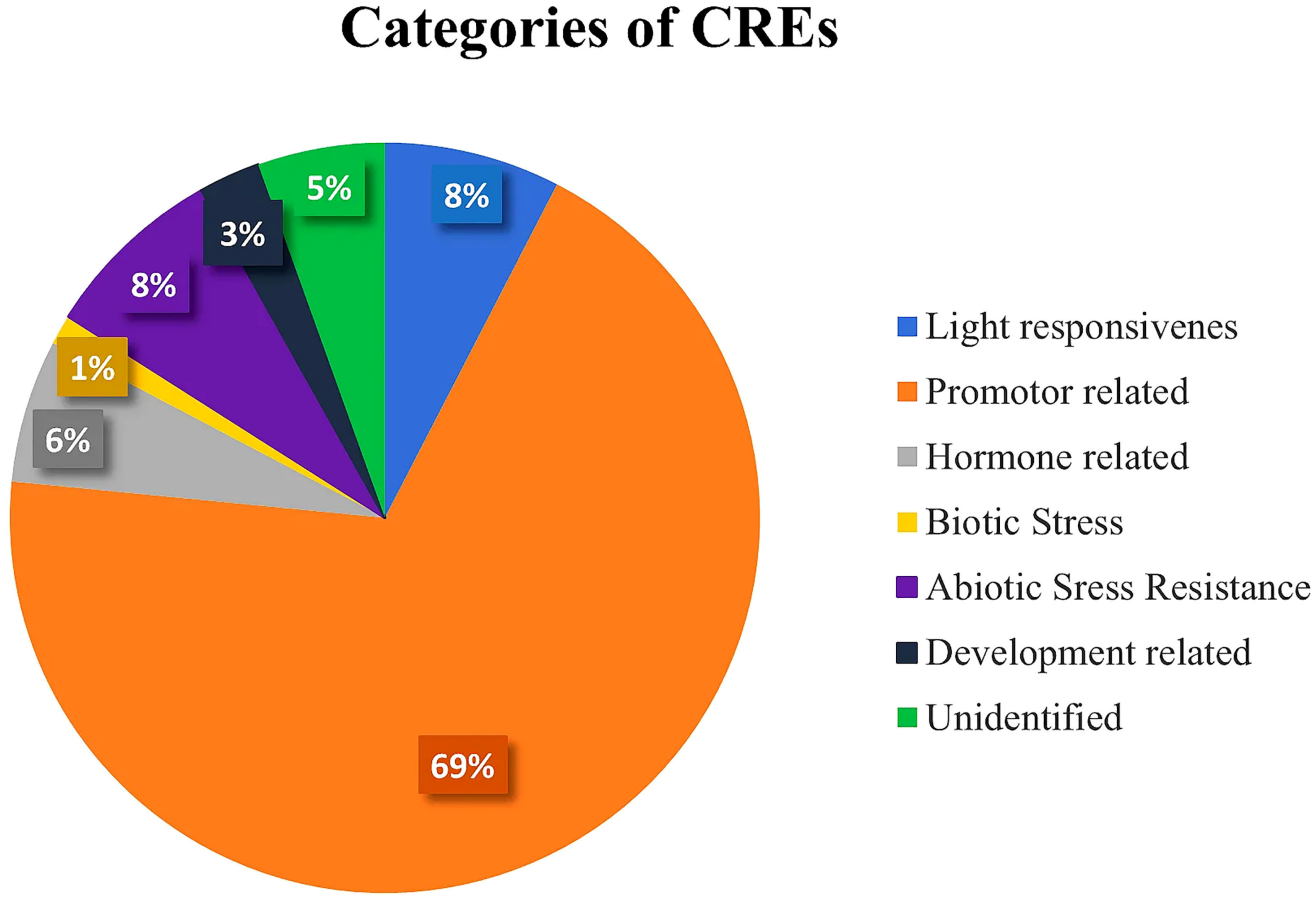
Pie chart representation of even categories of cis-regulatory elements with their respective percentages present in 58 SlDREB proteins.

Plant CARE program was employed on a 2,500-bp upstream region of 58 *Sl*DREB genes for the analysis of potential cis regulatory elements that are involved in the transcriptional activity and response to biotic and abiotic stresses in tomato. According to the analysis, 103 motifs were found and classified into seven different categories depending on the major functions performed by them (**Supplementary Table S7**). These classes were (i) promoter-related, (ii) light-responsive, (iii) abiotic stress-responsive, (iv) hormone-related, (v) development-related, (vi) biotic stress-responsive, and (vii) unidentified cis regulatory elements that were demonstrated through a statistical analysis. Promoter-related cis elements covered the largest area (69%), followed by light-responsive (8%), abiotic stress-responsive (8%), hormone-related (6%), unidentified cis elements (5%), development-related (3%), and biotic stress-responsive (1%), as shown in **Figure 9** (**Supplementary Table S7**).

**Figure 10:**
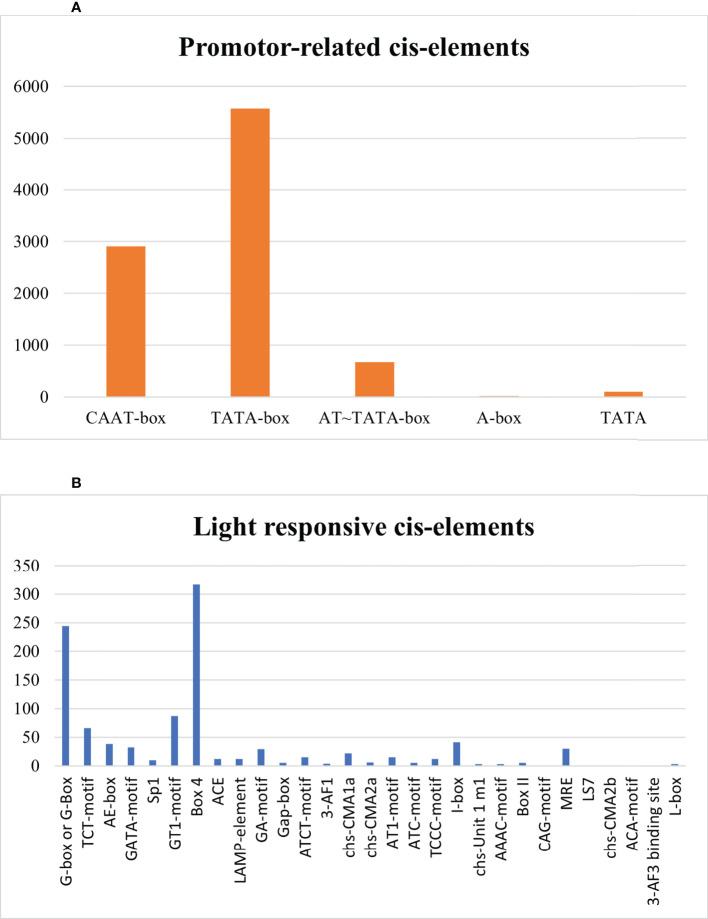
**(A)** Promoter-related cis regulatory elements. **(B)** Light-stress-responsive cis elements.

Assessment in *Sl*DREB proteins evaluated the presence of TATA-box, CAAT-box, AT~TATA box, A-box, and TATA elements that are involved in promoter binding site. TATA-box and CAAT-box were abundantly present in all 58 *Sl*DREB genes that start the transcription by acting as a binding site for transcription factors, whereas TATA element assists in the binding of TATA-box and initiation site, and AT–TATA box and A-box are promoter binding sites as illustrated in **Figure 10A**. Light-responsive category was comprised of G-box or G-Box, TCT-motif, AE-box, GATA-motif, Sp1, GT1-motif, Box 4, ACE, LAMP-element, GA-motif, Gap-box, ATCT-motif, 3-AF1, chs-CMA1a, chs-CMA2a, AT1-motif, ATC-motif, TCCC-motif, I-box, chs-Unit 1 m1, AAAC-motif, Box II, CAG-motif, MRE, LS7, chs-CMA2b, ACA-motif, 3-AF3 binding site, and L-box. Box4 and G-box motifs contained the large area that maintainsed the transcription rate of light-controlled genes (**Figure 10B**).

**Figure 11:**
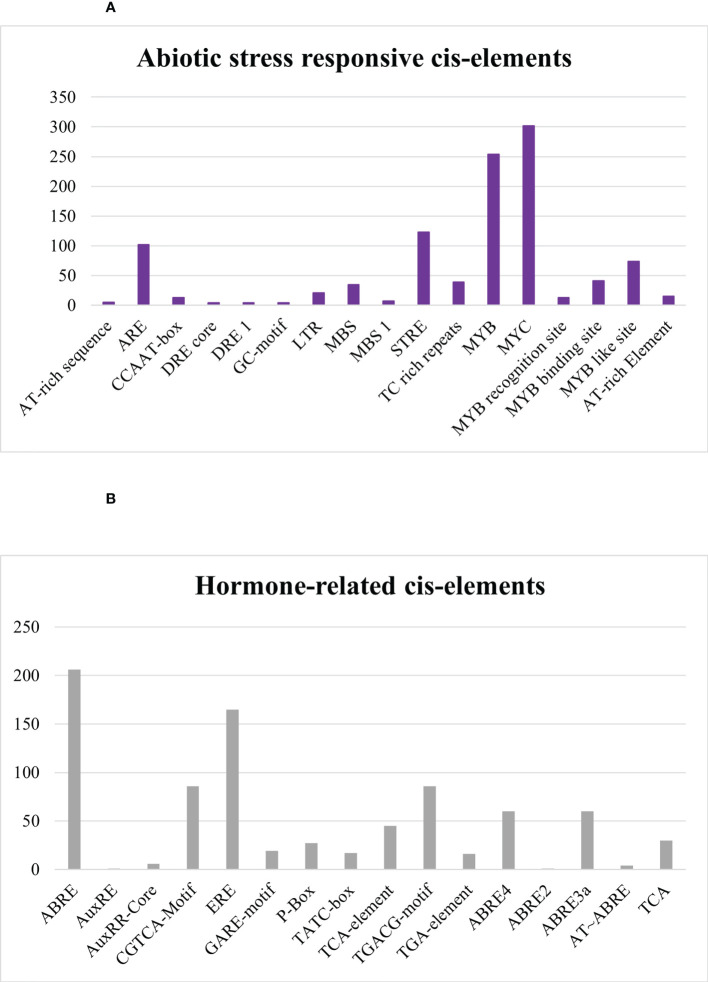
**(A)** Abiotic-stress-responsive cis regulatory elements. **(B)** Hormone-related cis regulatory elements.

In abiotic stress-responsive cis elements, the following motifs were revealed: AT-rich sequence, ARE, CCAAT-box, DRE core, DRE 1, GC-motif, LTR, MBS, MBS 1, STRE, TC rich repeats, MYB, MYC, MYB recognition site, MYB binding site, MYB like site, and AT-rich element as depicted in **Figure 11A**. Further results discovered ABRE, AuxRE, AuxRR-Core, CGTCA-Motif, ERE, GARE-motif, P-Box, TATC-box, TCA-element, TGACG-motif, TGA-element, ABRE4, ABRE2, ABRE3a, AT–ABRE, and TCA motifs in hormone-related cis element category. ABRE and ERE, also known as abscisic acid-responsive element and ethylene-responsive element, were greatly detected in *Sl*DREB genes in response to hormone-related changes that were further linked to abiotic stress like drought. DREB is a sub-family of ethylene-responsive factor (ERF) gene family, so ERE elements also play an important role in its regulation. Recently, extensive studies on ERF family were conducted in *Cucurbita moschata* (Li et al., 2022). ABRE4, ABRE2, ABRE3a, and AT–ABRE are abscisic acid-responsive elements and act as binding sites. AuxRE, TGA-element, and AuxRR-Core were involved in response to auxin, whereas CGTCA-Motif and TGACG-motif act as methyl jasmonate-responsive elements. TCA-element involved in salicylic response, P-box, TATC-box, and GARE-motif act as gibberellin-responsive elements as provided in **Figure 11B**, respectively.

**Figure 12:**
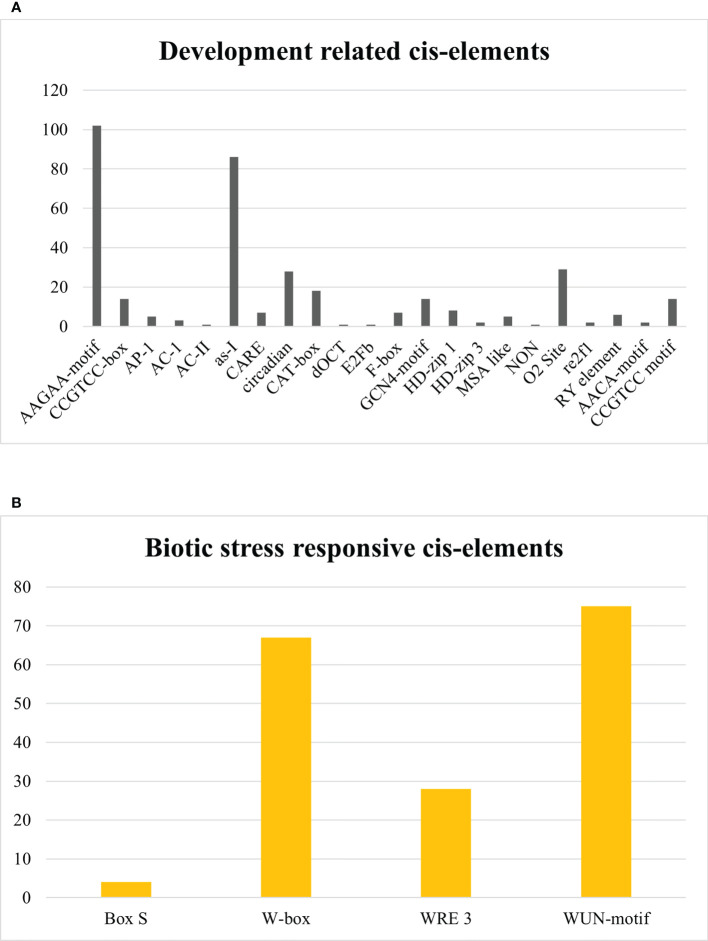
**(A)** Development-related cis regulatory elements. **(B)** Biotic-stress-responsive cis regulatory elements.

In development-related cis elements, AAGAA-motif, CCGTCC-box, AP-1, AC-1, AC-II, as-I, CARE, circadian, CAT-box, dOCT, E2Fb, F-box, GCN4-motif, HD-zip 1, HD-zip 3, MSA like, NON, O2 Site, re2f1, RY element, AACA-motif, and CCGTCC motif were found. AAGAA-motif and as-1 element, available abundantly, are pathogenesis-related promoters. Other development-related cis elements have a role in meristem-specific stimulation, xylem-specific activation, palisade mesophyll cell differentiation, vascular development, biosynthesis and degradation of biological macromolecules, endosperm expression element, regulation of circadian rhythms, regulation of zein metabolism, seed development, cell cycle regulation, and mitosis-specific activator functioning as given in **Figure 12A**. In contrast, Box S, W-box, WRE 3, and WUN-motif cis regulatory elements were recognized in the biotic stress-responsive category, which interact with WRKY transcription factors against biotic stresses and act as a wound-responsive element as depicted in **Figure 12B**. However, Unnamed:2, Unnamed:4, Unnamed:6, CTAG, Unnamed:1, Unnamed:8, Unnamed:10, Unnamed:12, Unnamed:14, and Box III were considered as motifs with unknown functions. A graphical illustration of all cis regulatory elements equivalent to *Sl*DREB genes is presented in **Supplementary Figure S8**. Identified cis elements might have an essential role in plant growth, development, and stress response.

### Secondary structure analysis and prediction of three-dimensional protein structure of SlDREB proteins

For protein modeling, five representatives (*Sl*DREB15, *Sl*DREB24, *Sl*DREB51, *Sl*DREB7, and *Sl*DREB54) from each class (A1, A2, A4, A5, and A6) were selected and subjected to SOPMA online server. The analysis exhibited that each selected protein had different percentages of α-helices, β turn, random coils, and extended strands. These variations in different secondary factors contributed to the versatility of protein functions, stability, effects protein sequences, interactions with other molecules, and structure. The α-helices, β turn, random coils, and extended strands were detected to be flanked by 24.55–50.31%, 3.76–5.45%, 3.88–59.27%, and 4.91–12.73% as shown in **Supplementary Table S9**, respectively.

The prediction of 3D protein model assists in the analysis functions, cellular location, and their interaction with other proteins by filling the gap between structure and original protein sequence. The 3D structure of selected *Sl*DREB proteins was predicted through Phyre2 server. Models with high percentage coverage (should be above 30%) and confidence were selected and subjected to further refinement for the construction of stable 3D structures.

### Structural validation, predicted catalytic sites, and disorder regions in *Sl*DREB proteins

For the structure refinement of *Sl*DREB proteins, retrieved models from the Phyre2 server were subjected to the Galaxy refine server. These refined models were submitted to the PROCHECK server that was utilized for the evaluation and quality of these refined models. About 90% of residues in every *Sl*DREB protein model were allowed in favorable regions when displayed in the Ramachandran plot that exhibited good stereo-chemical qualities of the generated model (**Supplementary Table S10**). Stable models were then visualized on ChimeraX software that exhibit a good quality of 3D structure with prominent α-helices and β turns represented in different colors (as depicted in **Supplementary Table S11**). To investigate the catalytic ligand binding sites in *Sl*DREB proteins, CASTp 3.0 server was used. It assists in predicting the location and representation of concave regions of active pocket sites which were illustrated in the orange region on the 3D structure of proteins as given in **Supplementary Table S12A**.

Besides the location, the results also confirmed the presence of many important amino acid residues in active catalytic sites that involved the functional versatility of proteins, including LYS, PHE, GLN, ALA, ASN, THR, PRO, HIS, MET, ILE, GLY, ASP, VAL, and ARG. The detailed surface area and volumes of catalytic sites were also given in **Supplementary Table S9**. The disorder regions of *Sl*DREB proteins were predicted using PrDOS software; it highlighted the disorder regions within the protein sequence as illustrated by the red regions (**Supplementary Table S12B**). The disorder regions had a significant role in the interactions and binding of proteins with other interactors, with high affinity depending on the cellular demand to cope with stresses or to regulate other metabolic pathways which was proved when the *Sl*DREB proteins were subjected to STRING for the analysis of functional associations.

### Interactive associations of *Sl*DREB proteins

To depict the *Sl*DREB protein associations with other proteins, STRING software, which provided 10 interactors associated with each *Sl*DREB protein according to the criteria were employed. The full STRING network indicates both the functional and structural associations of proteins, whereas the colored line represents the type of interaction. It illustrated the interactive networks, associated in distinct colors, of *Sl*DREB proteins with other proteins that belonged to different families, encoded by different genes, and contributed to the regulation of various biological and metabolic processes.

**Figure 13:**
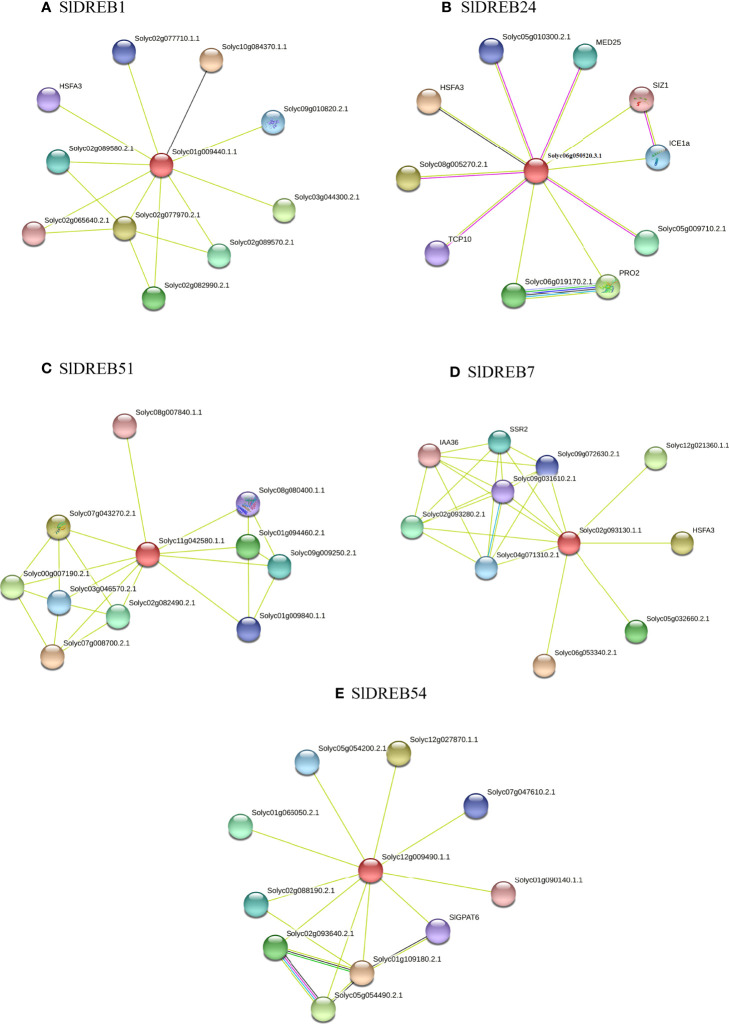
Interactive networks of SlDREB proteins through the STRING software. Protein interactions of SlDREB1 belong to A1 class denoted as **(A)**, SlDREB24 member of A2 as **(B)**, **(C)** SlDREB51, **(D)** SlDREB7, and **(E)** SlDREB 54 belong to A4, A5, and A6 classes, respectively. Colored networks depicted the associations depending on the information gathered from different databases, *i*.*e*., theoretical knowledge, experimental evidence, closely related genes, and protein model homology.

The interactive results of *Sl*DREB1, member of the A1 subgroup, demonstrated the interactions with Solyc10g084370.1.1, Solyc02g077970.2.1, Solyc03g044300.2.1, Solyc02g082990.2.1, Solyc02g089570.2.1, Solyc02g089580.2.1, Solyc09g010820.2.1, Solyc02g077710.1.1, HSFA3, and Solyc02g065640.2.1 as given in **Figure 13A**. The results confirmed that Solyc10g084370.1.1 and Solyc09g010820.2.1 showed the presence of the SANT domain which belongs to the homeobox superfamily and a MYB-related protein that acts as DNA binding domain and regulates histone acetylation. The LEA protein family was observed in Solyc02g077970.2.1, also described as late-embryogenesis abundant 76-like protein that is involved in the defense of plant against environmental stresses. In Solyc03g044300.2.1, the presence of the AP2 domain showed its function as DNA binding domain and contributed to the regulation of transcription factor activity and defense response. The KIN1 and KIN 2 domains were detected in Solyc02g082990.2.1, Solyc02g089570.2.1, Solyc02g089580.2.1, and Solyc02g065640.2.1, which confirmed these interactors as stress-induced proteins in response to cold and abscisic acid (ABA). It is also known as cor6.6 in *Arabidopsis*. E-6 like protein (Solyc02g077710.1.1) plays a crucial role in early fiber development, and heat shock factor A3 (HSFA3) was detected in Solyc09g009100.2.1 interactor, which was involved in response to heat, DNA binding, and regulation of transcription.

In *Sl*DREB24, which belonged to A2 class, this was connected with HSFA3, Solyc08g005270.2.1, PRO2, Solyc06g019170.2.1, Solyc05g009710.2.1, MED25, ICE1a, Solyc05g010300.2.1, TCP10, and SIZ1 (**Figure 13B**). The HSFA3 (Solyc09g009100.2.1) protein activates in response to heat stress. Solyc08g005270.2.1 contains the RST domain, which interacts with transcription factors and acts as regulator under stress, hormonal, and developmental response, whereas PRO2 (Solyc08g043170.2.1) and Solyc06g019170.2.1 is known as delta-1-pyrroline-5-carboxylate synthase, which regulates the biosynthesis of amino acids, osmoregulation, phosphorylation in cytoplasm, metabolic process, and proline biosynthesis. In Solyc05g009710.2.1, MED25 (Solyc12g070100.1.1), the mediator complex subunit25_von Willebrand factor type A, is present and acts as a positive regulator and scaffold protein for basal transcription factors and RNA polymerase II. ICE1a (Solyc06g068870.2.1), also known as an inducer of CBI expression1, is a DNA-binding protein present in the nucleus and induces cold tolerance. The RPOLD domain in Solyc05g010300.2.1 contributes to protein dimerization, DNA binding, and regulation of transcription. Moreover, TCP10 (Solyc07053410.2.1) is responsible in protein–protein interactions and DNA binding transcription factor (TF) activity and SIZ1 (Solyc11g069160.1.1), also known as SP-RING-type zinc finger domain that plays a crucial role in zinc ion binding activity and protein SUMOylating during signaling pathways.

Similarly, the potential interactors for *Sl*DREB51 (affiliated with A4 subgroup) were predicted and confirmed its associations with Solyc07g008700.2.1, Solyc07g043270.2.1, Solyc00g007190.2.1, Solyc01g094460.2.1, Solyc02g082490.2.1, Solyc09g009250.2.1, Solyc03g046570.2.1, Solyc01g009840.1.1, Solyc08g080400.1.1, and Solyc08g007840.1.1 as illustrated in **Figure 13C**. The FAR1-related sequence9 (Solyc07g008700.2.1) protein and Solyc07g043270.2.1 is a far-red elongated hypocotyl 3 isoform x involved in transcription regulation and circadian rhythms. The sigma factor PP2C-like phosphatases in Solyc00g007190.2.1 and Solyc02g082490.2.1 regulate protein dephosphorylation and serine/threonine phosphatase activity in the signaling pathway. Additionally, Solyc01g094460.2.1 contains AT-hook, and Solyc03g046570.2.1 (BES1-interacting MYC like protein) acts as a DNA-binding protein and controls protein dimerization activity. The Solyc01g009840.1.1 and Solyc08g080400.1.1 proteins belong to GRAS family that engaged in the gibberellin signaling pathway and are involved in plant growth and development. *Sl*DREB51 also interacts with Solyc08g007840.1.1 (*Sl*DREB34) to regulate the DNA binding transcription factor activity.

The STRING interactions predicted the association of the member of A5 subgroup (*Sl*DREB7) with Solyc06g053340.2.1, HSFA3, Solyc12g021360.1.1, Solyc05g032660.2.1, Solyc02g093280.2.1, SSR2, Solyc04g071310.2.1, Solyc09g072630.2.1, Solyc09g031610.2.1, and IAA36, as depicted in **Figure 13D**. Solyc06g053340.2.1 belongs to the bZIP (basic-leucine zipper domain) family, also known as F box, which is linked with SKP1 protein that is involved in transcriptional regulations. HSFA3 (Solyc09g009100.2.1) is a heat shock transcription factor A3 type which responds to a feverish temperature, whereas the UVR domain containing protein Solyc06g053340.2.1, short-chain dehydrogenases/reductases (SDR) (Solyc05g032660.2.1), acts in chlorophyl(ide) b reductase activity. The basic helix–loop–helix (bHLH) protein (Solyc02g093280.2.1) regulates the circadian rhythm, response to cold and light stress, carpel development, and protein dimerization. Moreover, succinic semialdehyde reductase isofom2 SSR2 (Solyc03g121720) is an RNA recognition motif domain. The CDK-activating kinase assembly factor-related Solyc04g071310.2.1 is associated with kinase activity, nucleotide excision repair, and positive regulations. Furthermore, the ROP-interactive CRIB motif-containing protein 7 (Solyc09g072630.2.1) is a protein binding domain. Finally, the damaged DNA-binding 2 protein (Solyc09g031610.2.1) and auxin-responsive protein (IAA36) (Solyc06g066020.2.1) control DNA repair mechanism, UV damage excision repair, protein poly-ubiquitination, response to UV-A and UV-B, transcription, and auxin-activated signaling pathway.

Correspondingly, *Sl*DREB54, a member of the A6 subgroup, displayed its connection with the Solyc01g109180.2.1 (long-chain acyl-CoA synthetase 2) which regulates beta-oxidation for the metabolism of lipid. Solyc12g027870.1.1 (cyclin-dependent kinase E1) acts as a catalytic domain, mediating phosphorylation and the kinase pathways. Solyc05g054490.2.1 is a member of 3-oxo-5-alpha-steroid 4-dehydrogenase family that is present in the membrane and involved in fatty acid biosynthesis. However, Solyc02g093640.2.1 is a SDR protein that is engaged in oxidoreductase activity. Solyc01g066050.2.1 is a transmembrane protein that belongs to tetratricopeptide repeat (TPR)-like superfamily protein present in the plasma membrane. Solyc02g088190.2.1 (SANT domain), Solyc05g054200.2.1, and Solyc07g047610.2.1 (transcriptional adapter ada2b isoform x1) respond to cold and are involved in histone acetylation and chromatin remodeling. *Sl*GPAT6 (Solyc09g014350.2.1) is a glycerol-3-phosphate 2-O-acyltransferase 6 protein detected in cutin biosynthesis, and Solyc01g090140.1.1 protein [belonging to 2-oxoglutarate (2OG) and Fe(II)-dependent oxygenase superfamily] is involved in the formation of plant hormones like pigments, flavonoids, gibberellins, and oxidoreductase activity, as presented in **Figure 13E**.

### Transcriptome analysis of *Sl*DREB genes

**Figure 14:**
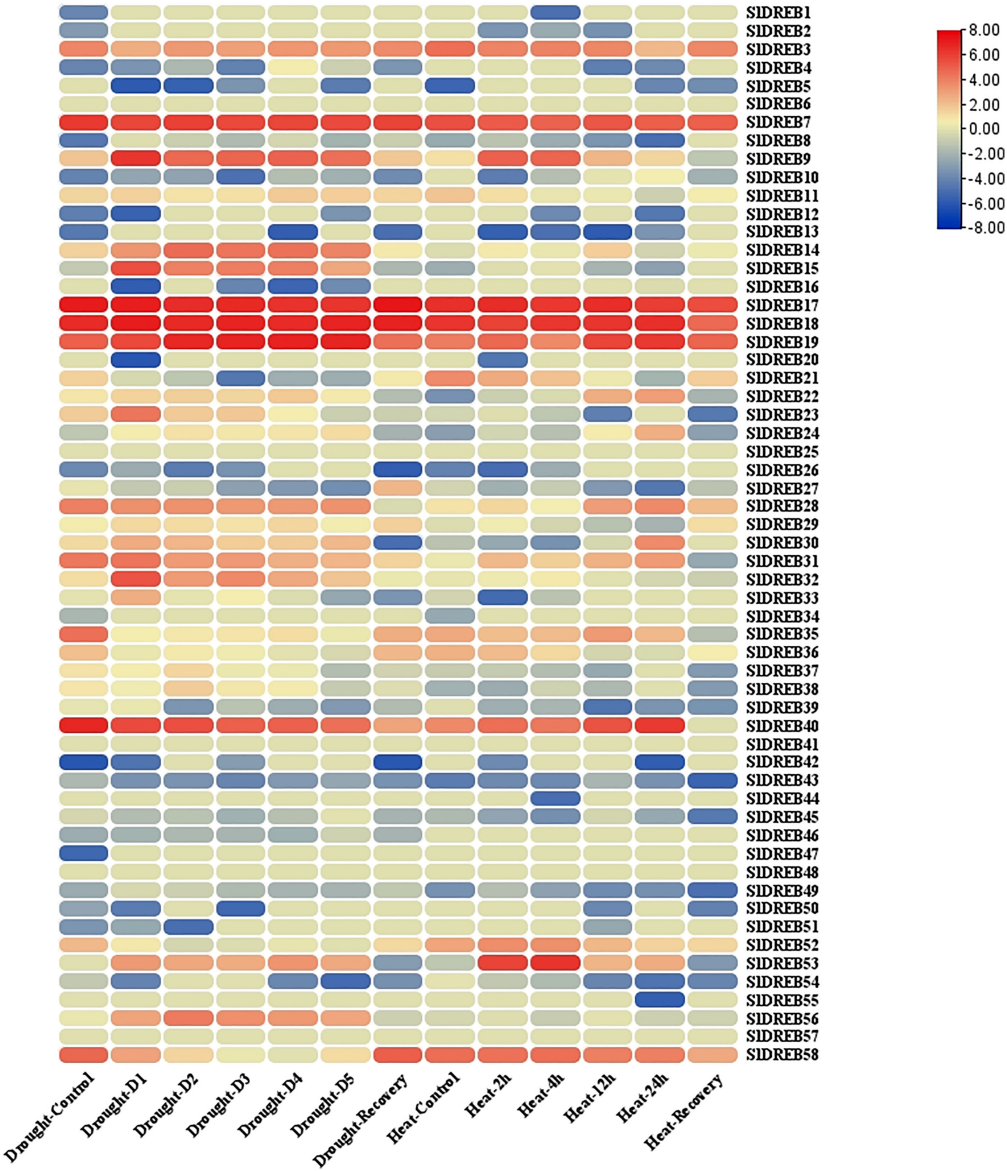
The expression analysis of 58 *Sl*DREB has been illustrated in the form of an expression heat map. Almost 29 *Sl*DREB genes were involved in response to drought and heat stress.

Transcriptomic data of tomato seedlings under drought and heat stresses was retrieved from the GEO database and further refined to extract *Sl*DREB expression levels (**Supplementary Table S13**). The mean values for similar sample sets were calculated to summarize the extensive datasets. Moreover, log_2_ base values were processed to quantify the expression levels of the respective genes (**Supplementary Table S13**). Furthermore, the expression levels were analyzed through an expression heat map (illustrated in **Figure 14**). The expression analysis of A1 sub-group demonstrates that *Sl*DREB8 and *Sl*DREB15 were upregulated under drought conditions and downregulated in response to heat stress. In addition, *Sl*DREB9 showed an increase in expression profiles in response to drought and heat stresses. Moreover, *Sl*DREB 32 and *Sl*DREB33 were upregulated in response to drought stress. However, *Sl*DREB1 indicated comparatively low expression profiles against both stresses, whereas *Sl*DREB57 did not respond to drought and heat stress treatments.

In A2 class, the transcriptomic expression of *Sl*DREB22 and *Sl*DREB24 was analyzed, which pointed towards a slight upregulation of both genes under drought stress; however, high expression profiles were reported in response to high temperature treatments. The results have shown that *Sl*DREB43 was downregulated in response to heat and drought stresses, although *Sl*DREB20 and *Sl*DREB44 did not respond to both stresses. Moreover, *Sl*DREB16 and *Sl*DREB46 were downregulated against drought and not involved in response to heat stress.

The transcriptome analysis of the A4 class revealed that *Sl*DREB36 highly responded towards both stresses. In addition, *Sl*DREB37 and *Sl*DREB838 were involved in defense signaling pathway and significantly expressed in response to water-deficient conditions, whereas *Sl*DREB39 was downregulated under stressful conditions. The results have demonstrated that *Sl*DREB3 was highly functional with significant expression profiles under water-deficient and high-temperature situations. Moreover, *Sl*DREB14 showed substantial expression levels under both stresses. Similarly, *Sl*DREB21 and *Sl*DREB28 were upregulated against drought and heat stress to activate the defense signaling pathway. However, in the case of *Sl*DREB34, *Sl*DREB41, *Sl*DREB48, *Sl*DREB50, and *Sl*DREB851, no significant expression profiles were indicated under stress conditions. However, high expression levels were reported for the *Sl*DREB52 gene, whereas *Sl*DREB56 was significantly upregulated in response to water-deficient conditions.

The transcriptome analysis data of A5 group members illustrated that *Sl*DREB26 and *Sl*DREB49 were downregulated whenever a plant undergoes heat and drought stresses. However, *Sl*DREB2 did not respond to both situations. Subsequently, *Sl*DREB7, *Sl*DREB30, and *Sl*DREB19 were highly expressive against drought stress. *Sl*DREB7 and *Sl*DREB9 were likewise upregulated in response to heat stress, whereas *Sl*DREB30 showed an insignificant expression level against heat stress. Furthermore, *Sl*DREB53 is involved in the drought and heat stress signaling pathway. The expression analysis of A6 class members revealed that *Sl*DREB11 showed elevated expression profiles under drought and high temperature. However, *Sl*DREB4, *Sl*DREB5, *Sl*DREB10, *Sl*DREB27, *Sl*DREB42, *Sl*DREB45, and *Sl*DREB54 demonstrate downregulation under both stresses, while *Sl*DREB17, *Sl*DREB18, and *Sl*DREB40 showed high expression levels against heat and drought stress. Moreover, *Sl*DREB25, *Sl*DREB47, and *Sl*DREB55 were not significantly responsive. On the other hand, *Sl*DREB58 and *Sl*DREB35 were highly expressed under heat stress and showed mild upregulation against drought stress. Lastly, *Sl*DREB31 was significantly upregulated against drought stress as compared to high temperature stress. These results conclude that DREB genes have an important role against drought and heat stress signaling in tomato.

## Discussion

The present study covers extensive data on 58 *Sl*DREB gene sequences. These 58 *Sl*DREB genes were classified into A1–A6 classes based on the classification of *At*DREB genes. The conserved amino acid analysis revealed that valine (V) is replaced by isoleucine (I) at the 14th position due to their same chemical structure, polarity, and characteristics in three *Sl*DREB genes. Both are aliphatic, non-polar, and hydrophobic, whereas glutamic acid (E) is being replaced by V, Q, D, H, L, and A at the 19th position (Zhang et al., 2022). The genome-wide analysis discovered the presence of 58 *Sl*DREB genes, which can vary in numbers in different plants due to the variance in genome sizes and genes present in their genome. The physiochemical properties, variation in amino acid structure, and protein binding affinity contribute multiplicity in protein function to withstand harsh environmental conditions (Salih et al., 2019; Panja et al., 2020). Thus, the variations in amino acids at conserved positions in *Sl*DREB genes proved that these genes regulate many biological and molecular mechanisms associated with plant growth and interact with other stress-regulatory proteins. The total length of *Sl*DREB proteins varied between 113 to 419 amino acids. Other properties like isoelectric point and molecular weight fluctuated between 4.42–9.41 and 12.35–46.94 kDa (**Supplementary Table S1**).

The phylogenetic analysis of *Sl*DREB and *At*DREB genes revealed that the variation in gene distribution patterns among A1–A6 classes, respectively, is due to genetic diversity. The study discovered the presence of seven members in A1 class and seven in A2 class, 16 members related to A4 class, seven members involved in A5, and 21 members present in A6 subgroup, respectively. It also demonstrated the absence of A3 class in the *Sl*DREB gene family because no *Sl*DREB member showed homology with AT2G40220.1 (member of A3 class) (**Figure 2**). Similarly, another related study reported the absence of A3 class in *Ananas comosus* (L.) Merr. (Chai et al., 2020). The research data illustrates that *Sl*DREB24 (Solyc06g050520, accession no: AF500011 or DREB1) has a significant role under drought stress (Jiang et al., 2017). *Sl*DREB22 (Solyc05g052410, GenBank accession: ADZ15315 or DREB2), known as *Sl*DREB2, is likewise a potential regulator of stress-responsive genes and is expressed in early-seedling stage (Hichri et al., 2016). SlDREB18 (Solyc04g072900) (GenBank accession: AF506825.1), named *Le*DREB3, showed enhanced tolerance against many abiotic stresses like drought, salinity, and osmotic pressure (Islam and Wang, 2009, Wang G. et al., 2019), and *Sl*DREB2 (Solyc06g066540) was recently characterized in response to heat stress (Mao et al., 2020). Furthermore, the gene structure analysis discovered the presence of introns in *S. lycopersicum*, unlike in *Arabidopsis.* The *At*DREB genes showed the lack of intronic regions in its coding sequence, but studies revealed the occurrence of introns in various plants like *S*. *tuberosum* (Mushtaq et al., 2021), soybean (Zhou et al., 2020), *Ananas comosus* (L.) Merr., and *Saccharum spontaneum* (Zhen et al., 2021). Studies revealed that 12 *Sl*DREB genes contain introns, out of which nine *Sl*DREBs had one intron and the remaining three *Sl*DREB genes entailed two introns. Thus, *S. lycopersicum* shows a similarity with *Ananas comosus*, *Solanum tuberosum*, *Saccharum spontaneum*, and soybean in terms of gene structure due to the presence of introns in the coding region. According to studies, gene structures with no or few introns are more active in response towards stress conditions (Jeffares et al., 2008). The difference in intron and exon organization clarifies the functional diversity of DREB genes that occurred due to variations in climatic conditions and stresses in a variety of plant species (Zhen et al., 2021).

To gain insight into the conserved motifs that have an important role in protein functionality and interactions, a motif analysis was accomplished. The results demonstrated the presence of conserved motifs in TFs on the same chromosome as in *Ananas comosus* (L.) Merr. and *Solanum melongena* L. (Li et al., 2021). In *Sl*DREB proteins, 20 conserved motifs were discovered, out of which two motifs were present throughout all *Sl*DREB sequences. It was observed that A1, A4, and A5 classes are more closely related, while A2 and A6 classes shared a combined clade due to the same conserved motifs. The AP2 domain consists of one alpha helix made up of 18 amino acids that assist in the interactions of DREB genes with other proteins, followed by three antiparallel beta sheets on the major groove of the DNA (Song et al., 2020). The identified 20 motifs in the *Sl*DREB sequences have a variable composition that is the possible reason for the classification of DREB genes into different classes with high structural resemblance with the AP2 domain. Motifs 1, 2, and 3 are specifically present in the AP2 domain, which is the characteristic domain of DREB genes, whereas a difference in motif composition evaluated the formation of two clades in the phylogenetic tree. The difference in motif composition is responsible for the molecular interactions with different proteins and assists in DNA binding (Alberts et al., 2002; Defoort et al., 2018). This study proved that a similar pattern of intron–exon organization and conserved motifs was observed in the members belonging to same groups, thus elucidating the reliability of the phylogenetic relationship represented in **Figure 3**.

The random distribution of *Sl*DREB genes provides an insight for their stability in a changing environment. Gene duplication events occurred in genes for adaptive evolution that is a major cause of plant diversity and adaptive specialization in genes (Flagel and Wendel, 2009). Polyploidy was observed in *Arabidopsis*, soybean, cotton, and potato (Nakano et al., 2006). Whole-genome duplication events occur in two ways: tandem duplication and segmental duplication. A tandem duplication event is responsible for the addition of a copied gene into the array, while segmental duplication is accountable for duplicating and spreading the fragments or the entire array. *S. spontaneum*, *Z. mays*, and *P. trichocarpa* go through both mechanisms of polyploidy. Whole-genome duplication was also examined in *S. lycopersicum*. Our results confirmed the occurrence of duplication events in 15 gene pairs, out of which eight gene pairs (32–33, 34–39, and 35–40) present on chromosome 08 (8–15, 27–29, and 50–51) exist on chromosomes 03, 06, and 11, and 43–44 and 45–47 belong to chromosome 10, undergoing tandem duplication events which were present on the same chromosomes (**Figure 4**).

In contrast, segmental duplication events were observed in seven gene pairs (36–52, 26–53, 11–54, 41–48, 17–58, 14–28, and 22–24) (**Figure 5**). In the tandem duplication method, gene pairs with the same cis regulatory elements, due to their presence on the same chromosome, were observed and involved in the regulation of similar functions (Flagel and Wendel, 2009). The present study’s data has revealed that the last gene duplication event occurred 4 million years ago in the 43–44 gene pair, which belongs to the A2 class and exhibits tandem duplication (**Supplementary Table S5**). Genes that are mostly involved in biological processes such as biotic and abiotic stress signaling undergo tandem duplications (Huang et al., 2020). To evaluate the presence of orthologous genes in interspecies, synteny analysis was employed. In total, 32 orthologs of *Sl*DREB genes were found in *Arabidopsis*, whereas 47 orthologs were observed in *S. tuberosum* due to the close evolutionary relationship with *S. lycopersicum*, with both belonging to the same family (**Figure 6**). For the analysis of functional genomics and molecular processes conducted by each *Sl*DREB gene, gene ontology annotation was employed. The results revealed that the DNA binding ability of *Sl*DREB genes is uniform, based on studies conducted in *P. vulgaris.* The GO annotation analysis also discovered that the *Sl*DREB genes primarily localized in the nucleus. Furthermore, the subcellular localization prediction showed the presence of *Sl*DREBs in 13 other organelles as illustrated in the heat map (**Figure 8**).

In this study, 103 CREs were found in *Sl*DREB sequences through promoter analysis. These 103 CREs have been classified into seven distinct categories depending on the functions and activation of the biological, cellular, and molecular pathways associated with them. Seven functional groups include light-responsive elements, hormone-related cis elements, development-related cis elements, biotic stress response elements, abiotic stress-responsive cis elements, promoter-related cis elements, and unidentified CREs (Ain-Ali et al., 2021). The promoter-related cis regulatory elements frequently occurred in all genes and secured about 69%, followed by light-responsive at 8%, abiotic stress-responsive at 8%, development-related at 6%, hormone-related at 3%, and biotic stress-responsive elements at 1% (**Figure 9**). The following cis elements are promoter-related: TATA-box, CAAT-box, AT~TATA box, A-box, and TATA elements. Among these elements, TATA-box and CAAT-box frequently occurred in every sequence and accommodated transcription factors by acting as their binding sites. The TATA element assists in the binding of TATA-box and activates the initiation site for transcription (Bae et al., 2015). The TATA box binds with RNA polymerase II for the initiation of transcription. AT~TATA box, and A-box are promoter binding sites. Moreover, A-box is an α-amylase promoter that is also identified in WRKY genes (Zhou et al., 2010). According to studies in rice, A-box assists in the regulation of the flowering stage (Mongkolsiriwatana et al., 2009). Thus, the analysis proved that *Sl*DREB genes have a strong role in transcriptional regulation (**Figure 10A**).

The cis regulatory elements involved in light response are G-box or G-Box, TCT-motif, AE-box, GATA-motif, Sp1, GT1-motif, Box 4, ACE, LAMP-element, GA-motif, Gap-box, ATCT-motif, 3-AF1, chs-CMA1a, chs-CMA2a, AT1-motif, ATC-motif, TCCC-motif, I-box, chs-Unit 1 m1, AAAC-motif, Box II, CAG-motif, MRE, LS7, chs-CMA2b, ACA-motif, 3-AF3 binding site, and L-box. G-box and Box-4 abundantly appeared and regulated the transcription of light-responsive genes (Gilmartin et al., 1990). Moreover, G-box, ACE, Sp1, and GATA-motif also regulate the responses against abiotic stresses (Shariatipour and Heidari, 2020). The TCT motif controls photoperiodism, which is crucial for circadian rhythms for the proper development of plants and for the flowering stage (Zhao et al., 2018). Studies have evaluated the involvement of G-box in the phosphorylation of light-responsive genes through the calcium pathway upon receiving light signal and upregulated rd29 in response to drought as well (Kurt and Filiz, 2018). It is also involved in ethylene and ABA signaling pathway by acting as a binding site for bZIP proteins and responds to many abiotic stresses such as UV light and hormonal changes (Ma et al., 2013; Saidi and Hajibarat, 2018). Box-4 is a highly conserved DNA module in genes that are lightly sensitive. WRKY genes are also highly concentrated with box-4 element (Yin et al., 2013). The GT-1 motif is involved in the upregulation of light responses (Wang C. et al., 2019). The same pattern of cis elements was observed in *S. tuberosum*, with the exception of 3-AF1 binding site, 3-AF3 binding site, chs-CMA2b, and ACA motif elements that are only specified in *S. lycopersicum* (**Figure 10B**). The light-responsive elements are highly associated with photoperiods that regulate circadian rhythms and many other processes like flowering stage (Ain-Ali et al., 2021).

Abiotic stress conditions severely affect the growth and development of tomato crop. AT-rich sequence, ARE, CCAAT-box, DRE core, DRE 1, GC-motif, LTR, MBS, MBS 1, STRE, TC-rich repeats, MYB, MYC, MYB recognition site, MYB binding site, MYB like site, and AT-rich element are associated with abiotic stress-responsive elements (**Figure 11A**). MYB and MYC are dehydration-responsive motifs mostly observed in *Sl*DREB gene promoters, whereas MBS and MBS1 act as binding sites for MYB that are engaged in drought response, biotic stress, and gene regulation of flavonoid biosynthesis (Li et al., 2015; Kaur et al., 2017). MYC is involved in the regulation of many hormone-related pathways. JA is one of the hormones mainly regulated through the MYC element in response to stresses (Yanfang et al., 2018). AT-rich elements act as binding site for AT-rich binding proteins and functions as activation mediator upon signal perception. Moreover, ARE is responsible for anaerobic induction and consists of two elements: GT and GC motif. GT motif has a high resemblance with MYB binding site and induces a response against anaerobic and water-deficient conditions (Dolferus et al., 2001). CCAAT-box acts as MYBHv1 binding site for heat shock factors against heat stress (Chaudhary et al., 2019). DRE core and DRE 1 are dehydration-responsive elements, whereas GC-motif is involved in anoxic-specific inducibility (Brown et al., 2003), LTR and STRE are low temperature stress-responsive elements, and TC-rich repeats are involved in defense signaling pathway (Lu et al., 2019; Niu et al., 2020). The cis elements associated with abiotic stress response appeared third in terms of occurrence and found to be consistent with prior studies conducted on *S. tuberosum* except the MYB like site.

Furthermore, among the results discovered were ABRE, AuxRE, AuxRR-Core, CGTCA-Motif, ERE, GARE-motif, P-Box, TATC-box, TCA-element, TGACG-motif, TGA-element, ABRE4, ABRE2, ABRE3a, AT~ABRE, and TCA motifs in the hormone-related cis elements category. Abscisic acid (ABA)-responsive element (ABRE) is associated with hormonal regulation and abiotic stress signaling. ABRE4, ABRE2, ABRE3a, and AT~ABRE act as binding sites (Verma et al., 2016). Another related study demonstrates that AuxRE, TGA, and AuxRR-Core cis elements are auxin-responsive and have a regulatory role in the expression of DREB genes under drought stress (Kolachevskaya et al., 2019). Moreover, auxin plays a significant role in plant development and defense signaling mechanisms against biotic and abiotic stresses, particularly in response to drought stress. Auxin, as a key growth hormone, assures the effective working of the plant physiology under stressful environments (Sharif et al., 2022), whereas CGTCA and TGACG-motif act as methyl jasmonate-responsive elements (Concha et al., 2013). Similar hormone-related elements were described in *Saccharum spontaneum* and *Solanum tuberosum* except ABRE4, ABRE2, ABRE3a, AT~ABRE, and TCA elements (Kaur et al., 2017). These hormone-related elements may have a direct or indirect interaction with signaling pathways in response to various stresses (**Figure 11B**).

In development-related cis elements, several motifs were discovered, such as AAGAA-motif, CCGTCC-box, AP-1, AC-1, AC-II, as-I, CARE, circadian, CAT-box, dOCT, E2Fb, F-box, GCN4-motif, HD-zip 1, HD-zip 3, MSA like, NON, O2 Site, re2f1, RY element, AACA-motif, and CCGTCC motif (**Figure 12A**). The AAGAA-motif and as-1 element, available abundantly, are pathogenesis-related promoters. The AAGAA-motif is responsible for the development of xylem and assists in water transportation. The As-1 element acts as a promoter binding site for many hormone-responsive genes, especially under auxin and methyl jasmonate stress signaling. The CCGTCC-box, CARE, and CAT-box act as cis elements in meristem-specific stimulation (Laloum et al., 2013). Moreover, AC-1 and AC-II are engaged in xylem-specific activation. HD-zip 1 and HD-zip 3 participate in palisade mesophyll cell differentiation and vascular development and regulate biological processes associated with macromolecules (Vulavala et al., 2017). AACA-motif and GCN4-motif act as endosperm expression elements and regulate the circadian rhythms (Kaur et al., 2017). O2 site is involved in the regulation of zein metabolism, and RY element assists in seed -specific regulation. However, E2Fb and F-box are engaged in cell cycle regulation, and MSA like is a mitosis-specific activator (Li L. et al., 2021). The present work demonstrates that S-box, W-box, WRE 3, and WUN-motif cis regulatory elements were recognized to function in response to biotic stresses and, furthermore, to interact with WRKY transcription factor which acts as a wound-responsive element as depicted in **Figure 12B** (Yin et al., 2013; Liu et al., 2016). According to another related study, W-box regulates seed dormancy and defense mechanisms against biotic stresses (Rinerson et al., 2015). These elements were also reported in *Saccharum spontaneum* and *Solanum tuberosum.* However, among 103 cis elements, 10 motifs which cover almost 5% occurrence were found with unidentified functions. Parallel studies revealed the existence of conserved motifs that might have a crucial role in many biological processes. Thus, the presence of these motifs in the promoter regions of *Sl*DREB genes indicates their potentialities in plant defense signaling and processes associated with growth and development in tomato.

Moreover, structural variations were detected in five *Sl*DREB representatives from each class through secondary and tertiary structure analysis, which gave insight to the development of stable protein structures. Secondary structures were created by SOPMA server that exhibited different percentages of α-helices, β turn, random coils, and extended strands. The spatial arrangement of amino acids present in the random coils of DREB proteins can vary depending on the intrinsic nature of protein. These factors contributed to the structural and functional versatility and molecular interactions of proteins (Vatansever et al., 2017). Furthermore, Phyre2 server assisted in the prediction of tertiary structures. Models that covered more than 30% coverage were further subjected for refinement. For structural refinement and validation, Galaxy Refine server and PROCHECK server were employed to complete the gaps between protein sequences and conformations to create stable protein structures and to check the stability through the generation of Ramachandran plots. Validation is considered as a core step in structural determination (Pražnikar et al., 2019). Studies reported that the residues lying from 85% to more than 90% in favored region is considered as a good model. Hence, this study indicated the presence of 91.2% for *Sl*DREB1, 88.8% for *Sl*DREB2, 89.4% for *Sl*DREB51, 90.3% for *Sl*DREB7, and 92.8% for *Sl*DREB54 residues present in the favored region. The percentages were represented through a Ramachandran plot that further verified the models having good stereo-chemical properties (**Supplementary Table S10**). Stabilized models were visualized through ChimeraX that constructed high-quality models with their respective α-helix and β turns (**Supplementary Table S11**).

There are specific regions on proteins that are responsible to interact with other proteins and molecules in response to different cellular and biological processes, and these are known as ligand binding sites (Kawabata, 2010). The present study data has revealed the presence of active catalytic sites on five *Sl*DREB protein structures that are crucial for binding with different proteins having variable binding capacities as indicated in orange region (**Supplementary Table S12A**). Each catalytic site is comprised of specific amino acids that contribute to the binding capacity, assortment in protein functions, interactions with other molecules, and structural versatility (Coleman and Sharp, 2010).

Furthermore, proteins consist of disorder regions that allow them to change their functions, expand their interactions, and induce structural versatility in response to the changing environment. These disorder regions are flexible parts that allow proteins to bind with greater affinity to different molecules and thus contribute to various defense signaling pathways and cell cycle processes. The disorder region analysis provides thorough understanding of the spatial regulations and interactions of signaling pathways associated with proteins. The presence of these flexible regions in transcription factors allow their binding with signaling cascades and other stress-responsive proteins (Pazos et al., 2013). Disorder regions were predicted in five representative *Sl*DREB proteins (**Supplementary Table S12B**). The binding plasticity of disorder regions assists in the attachment to other gene families like NAC, GRAS, and dehydrins. These interactions generate a signaling cascade in developmental processes like seed development, response to hormonal changes, and drought stress (Hsiao, 2022).

Protein–protein interactions are necessary for the regulation of different developmental and adaptive processes for normal plant growth (Braun et al., 2013). Transcription factors stimulate other proteins and co-factors for the activation of distinct regulatory pathways that control transcription in response to various stresses (Francois et al., 2020). In response to both biotic and abiotic stresses, thousands of molecules like reactive oxygen species (ROS), hormones, and proteins interact at the cellular and molecular levels for the activation of kinases and other signaling cascades. For the complete integration of protein function, its cellular abundance, and biochemical activities, it is crucial to gain insight to interactive pathways (Struk et al., 2019). The protein–protein interactions of five *Sl*DREB proteins were detected through STRING that revealed its physical and functional associations. In the present study (**Figure 13**), nodes indicate spliced isoforms and the interaction of query protein (red node) with different interactors due to post-translational modifications. Each node represents all the proteins produced by a single protein-coding gene locus. Moreover, colored edges represent the type of evidence in protein associations, blue and pink edges represent known interactions, and green, red, and dark blue colors are known for predicted interactions, whereas other colors indicate the evidence recovered from text mining or co-occurrence.

Moreover, our results demonstrate that *Sl*DREB1 interacts with 10 interactors, namely: Solyc10g084370.1.1, Solyc02g077970.2.1, Solyc03g044300.2.1, Solyc02g082990.2.1, Solyc02g089570.2.1, Solyc02g089580.2.1, Solyc09g010820.2.1, Solyc02g077710.1.1, HSFA3, and Solyc02g065640.2.1. Out of 10 interactors, two members (Solyc10g084370.1.1 and Solyc09g010820.2.1) showed the presence of SANT domain which belongs to homeobox superfamily and a MYB-related protein that acts as a DNA binding domain and regulates histone acetylation (Boyer et al., 2004). Histone acetylation is essential in the cell cycle and in response to abiotic stress (light, heat, UVB, sugar, salt, and wound) and hormonal change (salicylic, ethylene, abscisic acid and auxin) (Jiang et al., 2020). Histone acetyltransferase genes previously discovered in rice (Liu et al., 2012), *Triticum aestivum* (Gao et al., 2021), and *Arabidopsis thaliana* (Pandey et al., 2002) (Solyc02g077970.2.1) belong to the LEA protein family which are connected with salinity and water stress signaling and have been reported in many plants like *Vigna glabrescens* (Singh et al., 2022), cotton (Magwanga et al., 2018), *Salvia miltiorrhiza* (Chen et al., 2021), and *Triticum aestivum* (Liu et al., 2019). The presence of AP2 domain was detected in one member (Solyc03g044300.2.1) that contributed to transcriptional regulation and responded to stresses as found in wheat (Magar et al., 2022) and castor bean (Xu et al., 2013). However, four members of *Sl*DREB1 interactors—namely, Solyc02g082990.2.1, Solyc02g089570.2.1, Solyc02g089580.2.1, and Solyc02g065640.2.1—include the KIN1 and KIN2 domains, also known as cor6.6. in *Arabidopsis*, which is engaged in low temperature and responses to disturbance in abscisic acid levels, as abscisic acid is a vital hormone that activates in retort to stress stimuli (Wang et al., 1995). Solyc02g077710.1.1 is an E-6 like protein which is necessary for fiber development (John, 1996). Solyc09g009100.2.1 is the last interactor that is a heat shock transcription factor A3 (HSFA3) involved in response to heat stress, DNA binding, and regulation of transcription as illustrated in **Figure 13A**.

Several proteins were likewise associated with *Sl*DREB24, which belongs to the A2 subgroup, *i*.*e*., HSFA3, Solyc08g005270.2.1, PRO2, Solyc06g019170.2.1, Solyc05g009710.2.1, MED25, ICE1a, Solyc05g010300.2.1, TCP10, and SIZ1 (**Figure 13B**). Solyc08g005270.2.1 contains the RST domain which is present in PARP and TAF4s factors and belongs to the SRO gene family. It is engaged in transcriptional regulation by interacting with transcription factors and maintains ribosyl transferase activity. Ribosyl transferase activity is linked with oxidative stress and responsible for ADP-ribosyl polymerases (Jaspers et al., 2010). Extensive analysis of RST domain has been carried out in wheat (Liu et al., 2014), tomato (Zhen et al., 2021), banana (Zhang et al., 2019), *Sesamum indicum* (Liu et al., 2021), and rice (You et al., 2014), whereas two members [Solyc08g043170.2.1 (PRO2) and Solyc06g019170.2.1) are known as delta-1-pyrroline-5-carboxylate synthase. These members are associated with proline biosynthesis and osmoregulation. Proline synthesis is important in plants as it works as an antioxidant and signaling molecule under stress conditions, which further activates the defense signaling cascades. Furthermore, it controls osmotic pressure and ROS levels, thus making the plant tolerant to stresses (Hayat et al., 2012). In Solyc05g009710.2.1, MED25 (Solyc12g070100.1.1) mediator complex subunit25_von Willebrand factor type A was detected, which performs as a scaffold protein for transcription factors and RNA polymerase II and regulates hormonal cascades under biotic and abiotic stresses (Kazan, 2017). MED25 has been reported in *Arabidopsis*, where it is discovered to deal with drought stress (Elfving et al., 2011). Additionally, ICE1a (Solyc06g068870.2.1), also known as inducer of CBI expression1, is a DNA binding protein present in the nucleus and confers tolerance in plants against cold stress (Chinnusamy et al., 2003). The RPOLD domain in Solyc05g010300.2.1 contributes to protein dimerization and cellular response to elevated temperatures. Moreover, TCP10 and SIZ1 (SP-RING-type zinc finger domain), detected in Solyc07053410.2.1 and Solyc11g069160.1.1, are responsible for protein–protein interactions and DNA binding TF activity and play a crucial role in zinc ion binding activity and protein SUMOylating. Studies in rice revealed that protein SUMOylation is a process in which local proteins act as signal transducers to activate signal cascades under stress (Raorane et al., 2013).

*Sl*DREB51 likewise belongs to the A4 class. It shows interactions with Solyc07g008700.2.1, Solyc07g043270.2.1, Solyc00g007190.2.1, Solyc01g094460.2.1, Solyc02g082490.2.1, Solyc09g009250.2.1, Solyc03g046570.2.1, Solyc01g009840.1.1, Solyc08g080400.1.1, and Solyc08g007840.1.1 (**Figure 13C**). FAR1-related sequence9 (Solyc07g008700.2.1) protein and Solyc07g043270.2.1 are a far-red elongated hypocotyl 3 isoform x involved in transcription regulation, response to hormonal change, and light stress. Other functions that are revealed in other plants are the biosynthesis of chlorophyll and starch and circadian rhythms (Ma and Li, 2018). The sigma factor PP2C-like phosphatases in Solyc00g007190.2.1 and Solyc02g082490.2.1 regulate protein dephosphorylation and serine/threonine phosphatase activity in the signaling pathway. Additionally, Solyc01g094460.2.1 contains AT-hook and DUF 296 domain which is a domain of unknown function, and Solyc03g046570.2.1 (BES1-interacting MYC like protein) acts as a DNA binding protein and controls protein dimerization activity. *Sl*DREB51 also interacts with Solyc08g007840.1.1 (*Sl*DREB34), which demonstrated that *Sl*DREB members are associated with each other for the regulation of DNA binding activity and response to biotic and abiotic stresses. The Solyc01g009840.1.1 and Solyc08g080400.1.1 proteins belong to the GRAS family, which is engaged in gibberellin signaling pathway and functions in plant growth and development. The GRAS family has been analyzed in various plants like rice, *Populus* and *Arabidopsis* (Liu and Widmer, 2014), *Glycine max* (Wang et al., 2020), *Prunus mume* (Lu et al., 2015), and tomato (Huang et al., 2015).

The present study has established that *Sl*DREB7 is A5 class member and associated with the following proteins: Solyc06g053340.2.1, HSFA3, Solyc12g021360.1.1, Solyc05g032660.2.1, Solyc02g093280.2.1, SSR2, Solyc04g071310.2.1, Solyc09g072630.2.1, Solyc09g031610.2.1, and IAA36 (**Figure 13D**). One member (Solyc06g053340.2.1) belongs to the bZIP (basic-leucine zipper domain) family which is a prominent TF family associated with stress signaling pathways and developmental processes. In addition, studies on *Arabidopsis* and potato have revealed its functions in senescence, light and biotic stress response, and seed growth (Wang et al., 2021). Solyc06g053340.2.1 is a UVR domain-containing protein, and Solyc05g032660.2.1 is SDR, which shows oxidoreductase activity. SDR is of a heterogenous protein family which is involved in secondary metabolic and developmental pathways such as catabolism and flower growth (Moummou et al., 2012). However, Solyc02g093280.2.1 is recognized as bHLH DNA-binding protein that controls circadian rhythm, response to cold and light stress, carpel development, and protein dimerization. bHLH is a significant transcription factor family which is discovered in several plant species, *i*.*e*., *Capsicum annuum L*. (Zhang et al., 2020), tomato (Sun et al., 2015), and maize (Li et al., 2018). Moreover, succinic semialdehyde reductase isofom2 SSR2, recognized in Solyc03g121720, is an RNA recognition motif domain, and Solyc04g071310.2.1 is a CDK-activating kinase assembly factor-related protein that is associated with kinase activity and nucleotide excision repair, whereas ROP-interactive CRIB motif-containing protein 7 (Solyc09g072630.2.1) belongs to the Rho family that reacts to oxygen deficiency, cellular development, and hormone-mediated pathways (Yang and Fu, 2007). Finally, damaged DNA-binding 2 protein (Solyc09g031610.2.1) and auxin-responsive protein (IAA36) (Solyc06g066020.2.1) control the DNA repair mechanism, UV damage excision repair, protein poly-ubiquitination, response to UV-A and B, transcription, and auxin-activated signaling pathway (Reed, 2001).

STRING interactions revealed the network of *Sl*DREB54 (member of the A6 subgroup) with Solyc01g109180.2.1 (long-chain acyl-CoA synthetase 2) which regulates and controls beta-oxidation for lipid metabolism. The second member is Solyc12g027870.1.1, a cyclin-dependent kinase E1 (CDKE1) that acts as a catalytic domain, mediating phosphorylation and the kinase pathways. In a study, CDKs revealed their functions in cell cycle regulations (Malumbres, 2014). Solyc05g054490.2.1 belongs to the 3-oxo-5-alpha-steroid 4-dehydrogenase family protein that functions in fatty acid biosynthesis. However, Solyc02g093640.2.1 is a fourth interactor (SDR protein) engaged in oxidoreductase activity. Solyc01g066050.2.1 (fifth interactor) is a transmembrane protein that belongs to the TPR-like superfamily present in the plasma membrane. The TPR family is involved in phytohormone signaling like abscisic acid, cytokinins, and gibberellins and also regulates ethylene signaling pathways. Furthermore, it regulates biological processes under osmotic stress conditions in plants (Schapire et al., 2006), and Solyc02g088190.2.1 consists of a SANT domain.

Moreover, Solyc05g054200.2.1 and Solyc07g047610.2.1 (transcriptional adapter ada2b isoform x1), associated with cold stress signaling, are involved in histone acetylation and chromatin remodeling. *Sl*GPAT6 (Solyc09g014350.2.1) is a glycerol-3-phosphate 2-O-acyltransferase 6 protein detected in cutin biosynthesis in tomato (Petit et al., 2021). Additionally, *Sl*DREB54 revealed its interaction with Solyc01g090140.1.1 protein [belongs to 2-oxoglutarate (2OG) and Fe(II)-dependent oxygenase superfamily] that functions in the biosynthesis of plant pigments, flavonoids, and gibberellins and regulates oxidoreductase activity (Kawai et al., 2014) (**Figure 13E**). The transcriptome analysis of tomato predicted that about 29 *Sl*DREB genes are actively involved in regulating the defense pathways against abiotic stresses such as drought and heat stress. Only four DREB genes have already been characterized through an expression analysis that revealed their significant role against abiotic stresses. Recent studies conducted on *Sl*DREB24 (Solyc06g050520, accession no: AF500011 or DREB1) showed its remarkable role in response to drought stress. It enhances the sugar concentration and accumulates osmolytes that further normalize the osmotic pressure and increase the response to drought stress (Jiang et al., 2017). *Sl*DREB22 (Solyc05g052410, GenBank accession: ADZ15315 or DREB2), known as *Sl*DREB2, is also a potential regulator of stress-responsive genes. According to spatiotemporal expression, this gene is also induced during early-seedling stage and is present in the vegetative parts of a plant. It is highly expressed in the leaves in defense against drought and salt stress (Hichri et al., 2016). Consequently, *Sl*DREB18 (Solyc04g072900; GenBank accession: AF506825.1), named *Le*DREB3, has been reported to enhance the tolerance against many abiotic stresses like drought, salinity, and osmotic pressure. It is also known as the negative regulator of the ABA pathway, thus affecting many developmental processes like germination and senescence. It is also engaged in many physiological processes like root architecture development (Islam and Wang, 2009; Wang G. et al., 2019). Similar studies also revealed the expression of *Sl*DREB28 (Solyc06g066540) under heat stress. It functions in response to many biotic and abiotic stresses such as salinity, drought by a fluctuating hormone level that contributes to increasing tolerance against stress. It assists in the biosynthesis of phytohormones (jasmonic acid, salicylic acid, and ethylene) and binds to DRE element under stress conditions (Mao et al., 2020). Other genes that were downregulated or not expressed under heat and drought stress might be involved in other abiotic stresses, developmental mechanisms, and the regulation of several stress-resistant genes involved in biotic responses. Previous studies on expression analysis exhibit the role of DREB genes in fruit development and ripening (Zhang et al., 2022). Overall, the present study data illustrates the comprehensive interactive networks of *Sl*DREBs with various other stress-responsive proteins associated with the regulation of development, growth, and stress tolerance processes in tomato.

## Conclusion

In conclusion, our results revealed a total of 58 *Sl*DREB genes in tomato, with the presence of a conserved AP2 domain. Only three DREB genes indicated the replacement of valine with isoleucine due to the same polarity, structure, and chemical characteristics. Multiple sequence alignment and phylogenetic evaluation demonstrated the classification of *Sl*DREB genes into five subgroups, excluding the A3 class, unlike *Arabidopsis*, which comprised A1–A6 classes. Gene duplication events proved the occurrence of polyploidy by tandem and segmental duplication methods. The evaluation of (Ka/Ks) values confirmed the existence of strong purification selection during the evolution of plants by calculating the divergence time. Moreover, synteny analysis revealed 32 and 47 orthologs in *Arabidopsis* and *Solanum tuberosum*, respectively. The cis-regulatory element analysis discovered 103 motifs that were further divided into seven categories. These elements have been implicated in light response, hormonal modifications, development, abiotic stress-responsive, biotic stress, and promoter binding sites. Gene ontology data illustrated that the *Sl*DREB genes are involved in various biological and molecular processes. A subcellular localization assessment confirmed that the *Sl*DREB genes primarily resided in the nucleus and were observed in 13 other organelles in variable concentrations. The secondary structure analysis assisted in determining essential points, whereas the tertiary structures of *Sl*DREB proteins and validation of protein models provide a thorough understanding of homology modeling. The results of active ligand binding sites and disordered regions provide a deep insight into the protein’s structural and functional plasticity and versatility. Moreover, interactive network analysis demonstrated the connections of *Sl*DREB proteins with other potential interactors. These interactions result in extensive signaling pathways and the functional diversity of *Sl*DREB proteins. Transcriptome analysis revealed that 29 *Sl*DREB genes regulate the defense signaling mechanisms against drought and heat stress. Overall, our data has potential information that serves as a robust infrastructure for unravelling the extensive structural and functional relationships of intricate genes requisite for genetic engineering, advance breeding, and biogenetics to develop stress tolerant/over-expressive tomato genotypes.

## Data availability statement

The datasets presented in this study can be found in online repositories. The names of the repository/repositories and accession number(s) can be found in the article/**Supplementary Material**.

## Author contributions

FM designed the project and scheme of work. HM and FM conducted the research, analyzed the data, and wrote the manuscript. FM, RA, and AG reviewed the manuscript. All authors contributed to the article and approved the submitted version.

## Acknowledgments

We acknowledge the Higher Education Commission, Pakistan, and the National University of Sciences and Technology (NUST), Pakistan, for providing research facilities.

## Conflict of interest

The authors declare that the research was conducted in the absence of any commercial or financial relationships that could be construed as a potential conflict of interest.

## Publisher’s note

All claims expressed in this article are solely those of the authors and do not necessarily represent those of their affiliated organizations, or those of the publisher, the editors and the reviewers. Any product that may be evaluated in this article, or claim that may be made by its manufacturer, is not guaranteed or endorsed by the publisher.
