# Supplementary material for: Genome-wide identification, comprehensive characterization of transcription factors, cis-regulatory elements, protein homology, and protein interaction network of DREB gene family in Solanum lycopersicum
Source: Front Plant Sci. 2022 Nov 24;13:1031679. doi: 10.3389/fpls.2022.1031679 (PMC9731513; doi:10.3389/fpls.2022.1031679)
Supplement: Supplementary file 4 [file Table_4.docx]

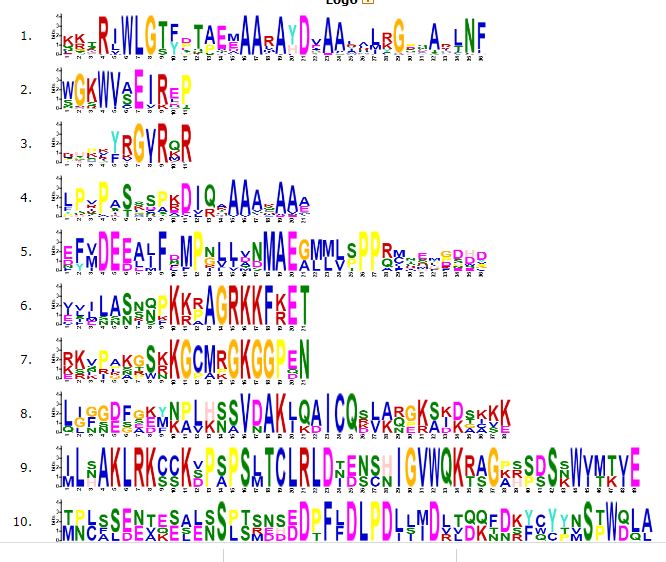


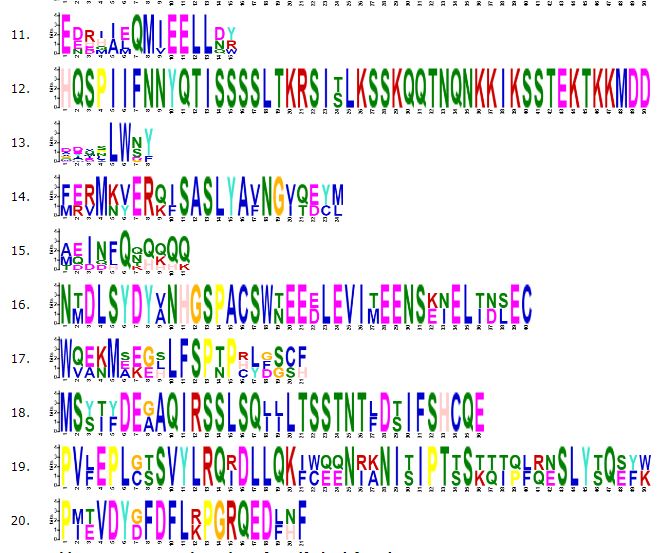


**Supplementary Figure :** Motifs revealed in 5 classes of *Sl*DREB proteins. Height of the motif represents the frequency of abundance.
