# Supplementary material for: Genome-wide identification, comprehensive characterization of transcription factors, cis-regulatory elements, protein homology, and protein interaction network of DREB gene family in Solanum lycopersicum
Source: Front Plant Sci. 2022 Nov 24;13:1031679. doi: 10.3389/fpls.2022.1031679 (PMC9731513; doi:10.3389/fpls.2022.1031679)
Supplement: Supplementary file 8 [file Table_8.docx]

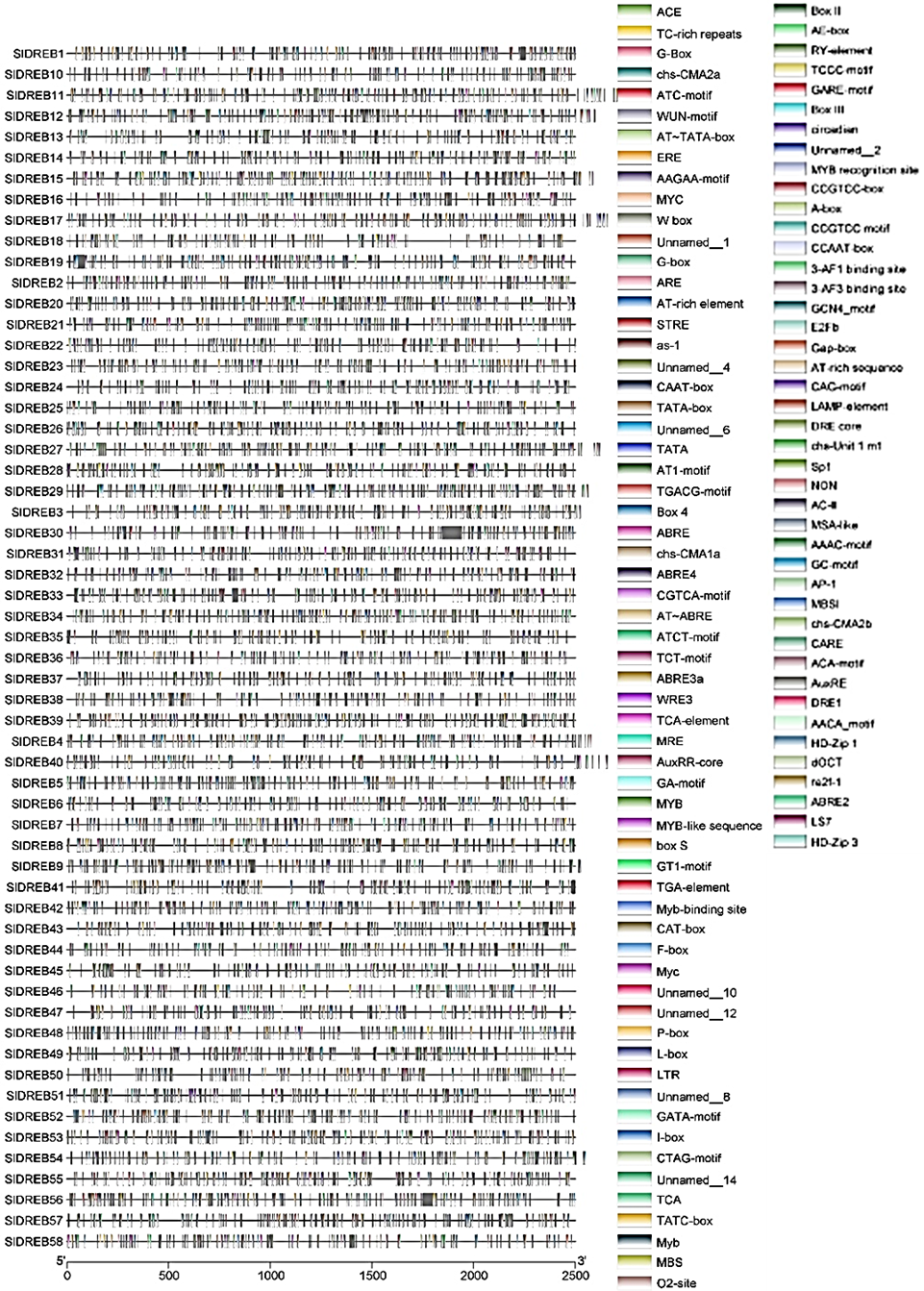


**Supplementary Figure:** Cis regulatory elements (CREs) analysis in 58 SlDREB sequences. Each bar indicates specific CRE which is designated with specific color.
