# Supplementary material for: Genome-wide identification, comprehensive characterization of transcription factors, cis-regulatory elements, protein homology, and protein interaction network of DREB gene family in Solanum lycopersicum
Source: Front Plant Sci. 2022 Nov 24;13:1031679. doi: 10.3389/fpls.2022.1031679 (PMC9731513; doi:10.3389/fpls.2022.1031679)
Supplement: Supplementary file 10 [file Table_10.docx]

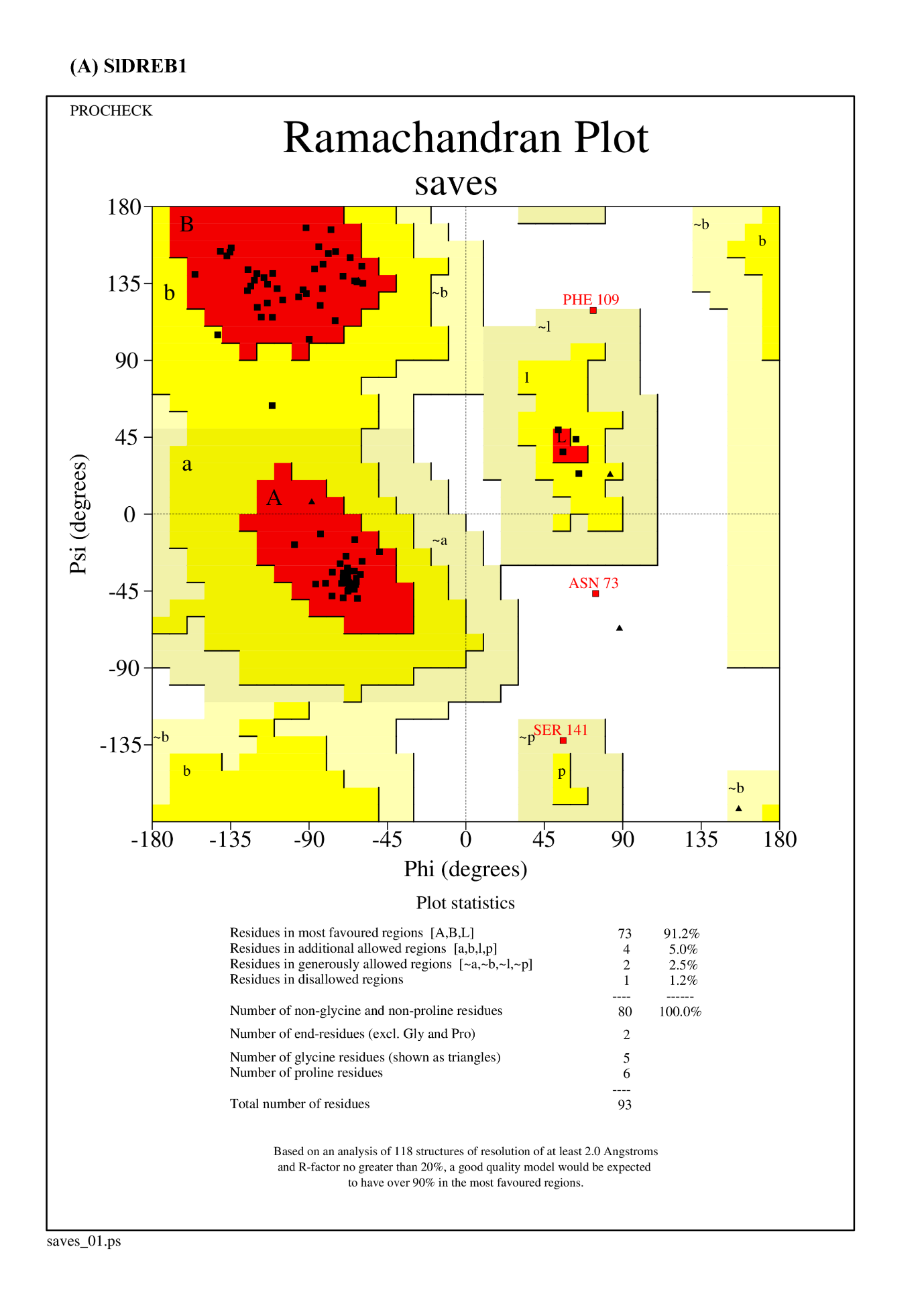


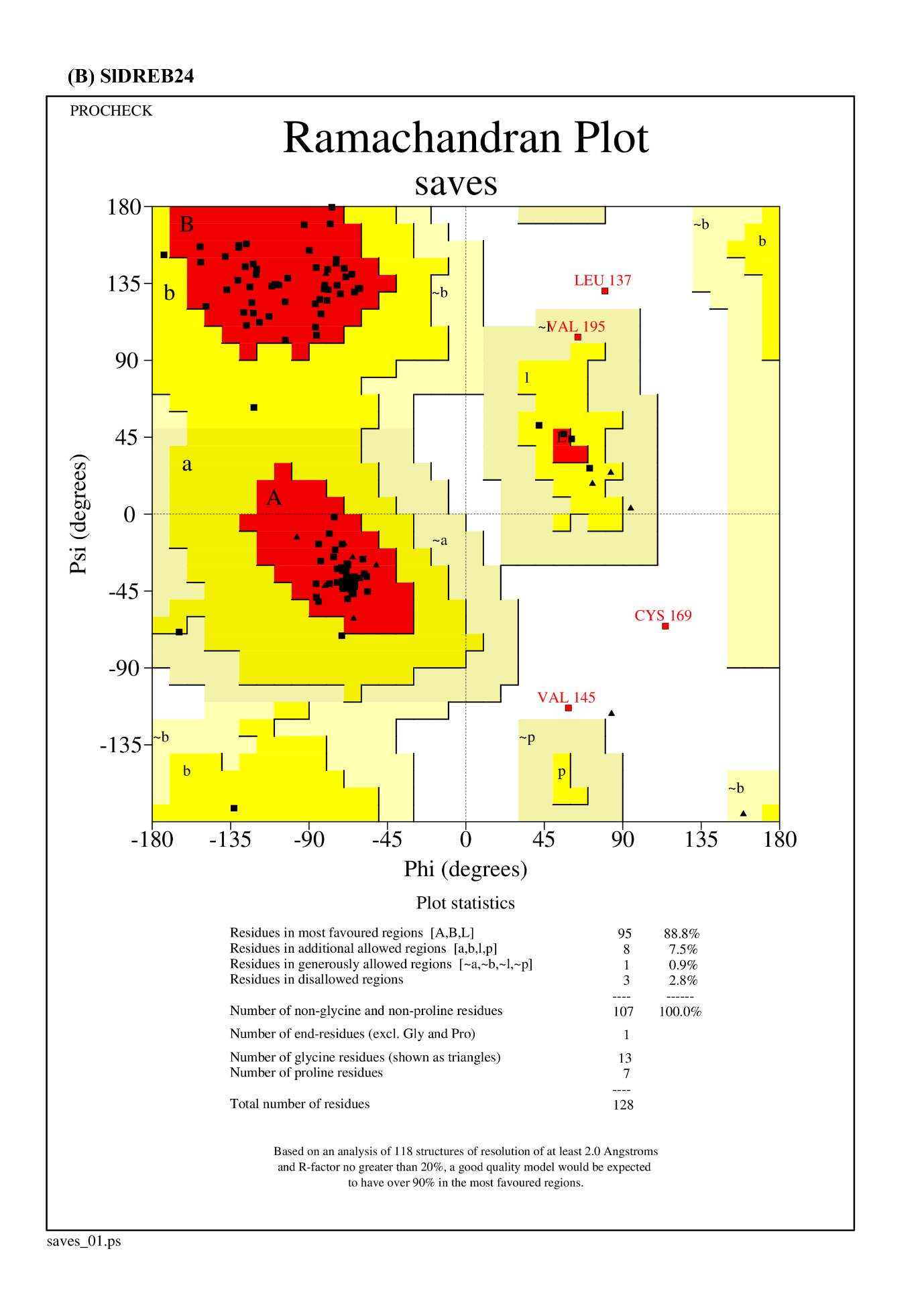


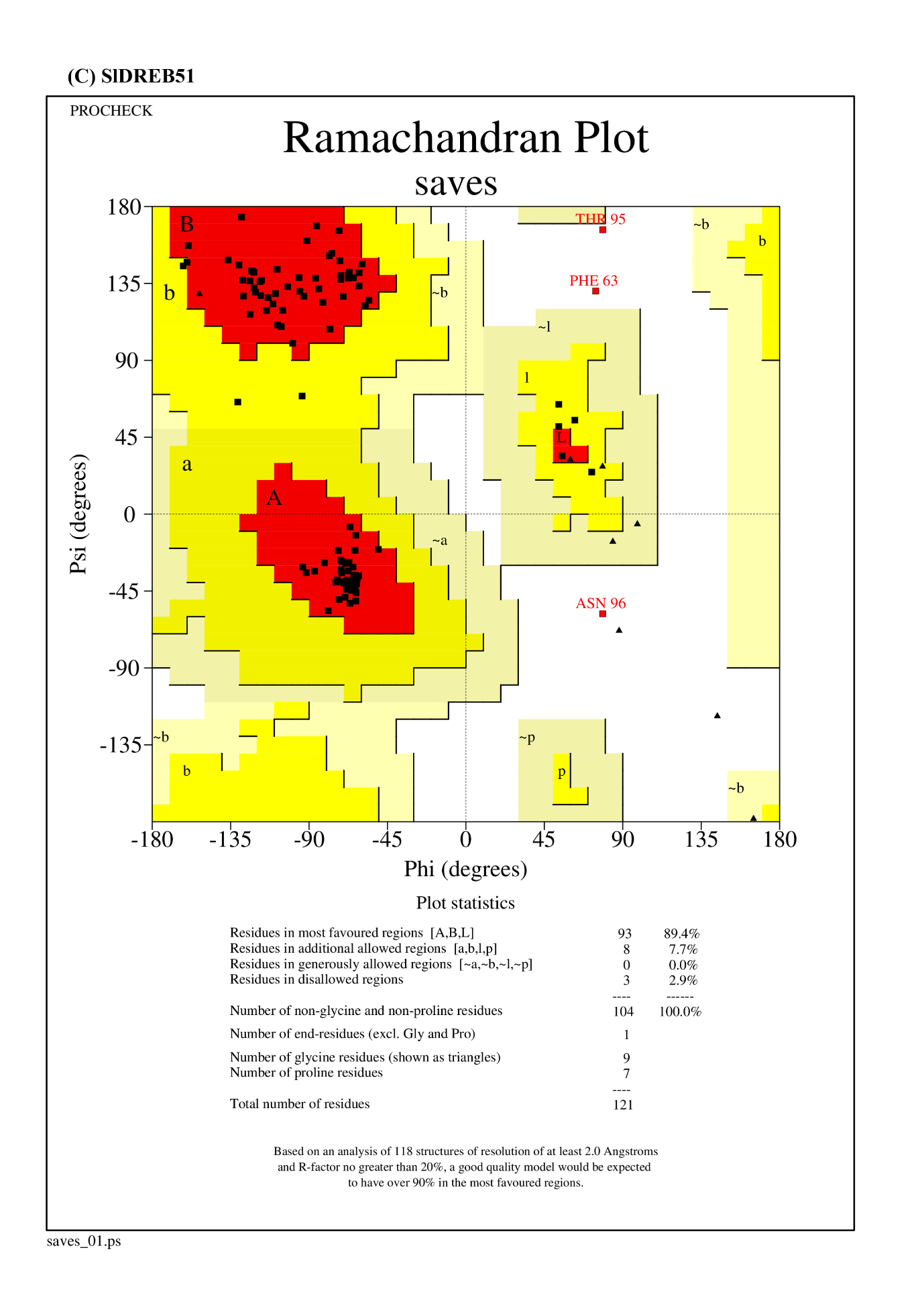

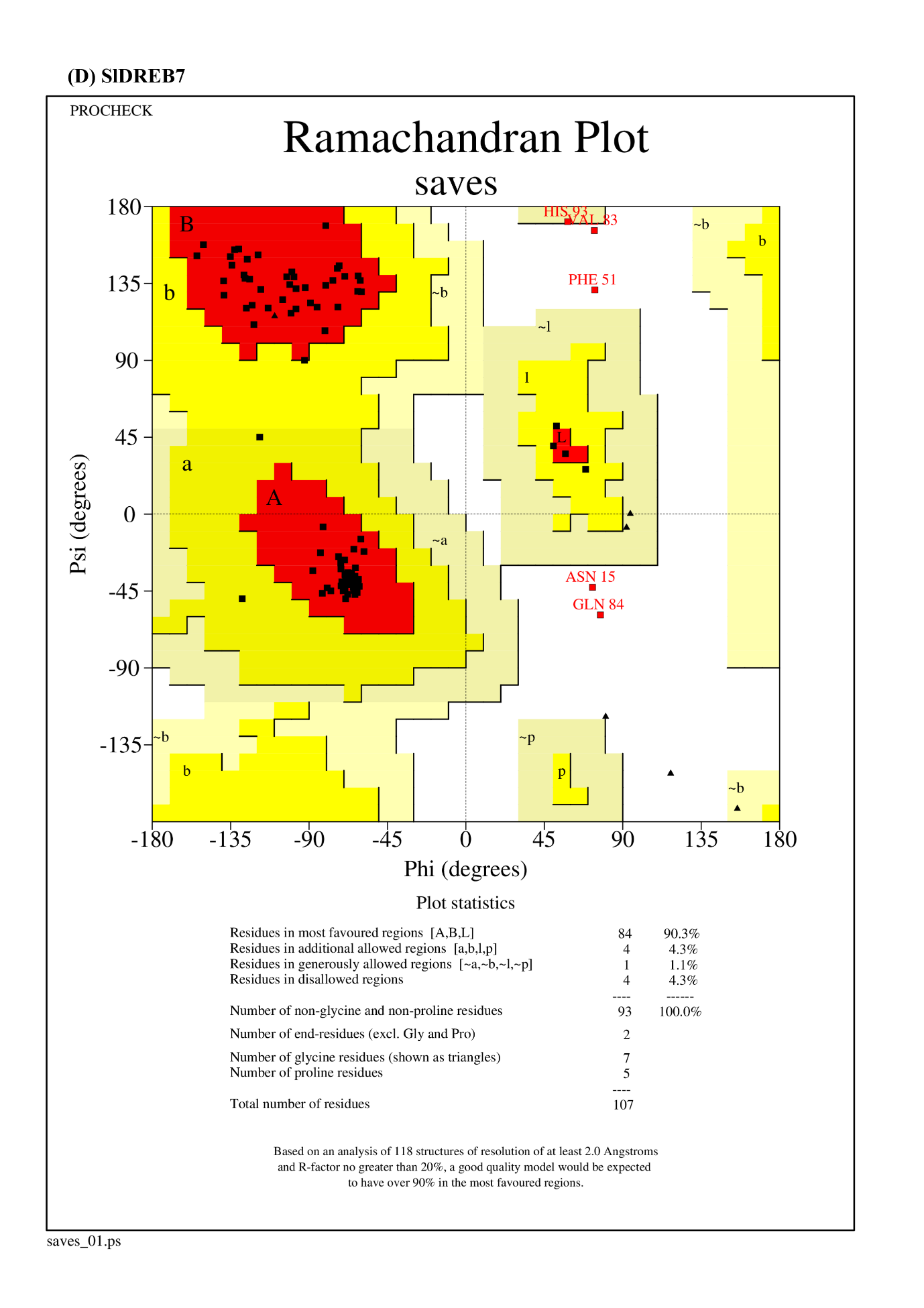

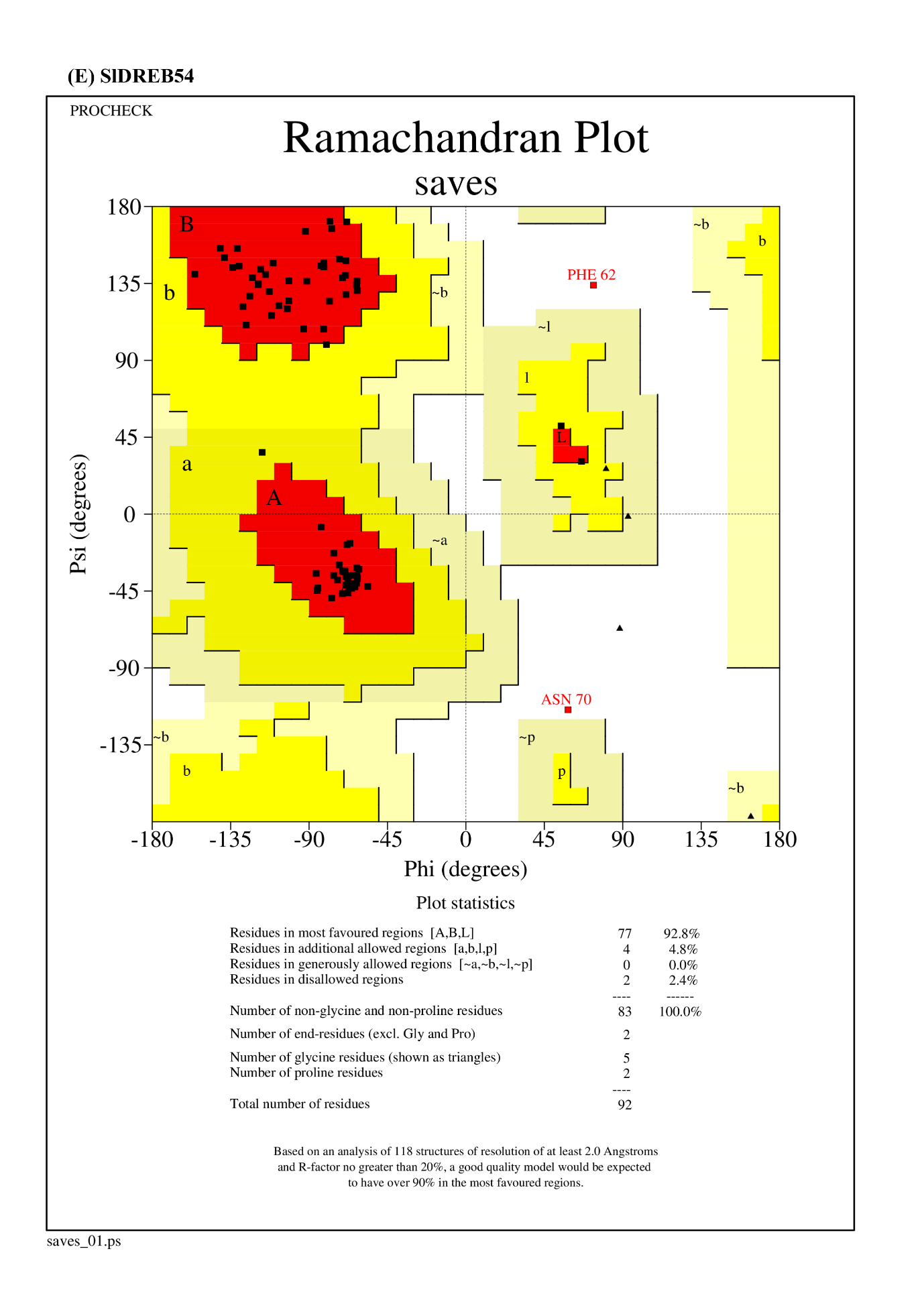


**Supplementary Figure**: Ramachandran plots of five representatives of SlDREB proteins through ProCheck Server. The plots of (A) SlDREB1, (B) SlDREB24, (C) SlDREB51, (D) SlDREB7, (E) SlDREB54 depicted 90% residues in favorable regions which indicate that selected protein models are stable after refinement through Galaxy Refine Server.
