# Supplementary material for: Genome-wide identification, comprehensive characterization of transcription factors, cis-regulatory elements, protein homology, and protein interaction network of DREB gene family in Solanum lycopersicum
Source: Front Plant Sci. 2022 Nov 24;13:1031679. doi: 10.3389/fpls.2022.1031679 (PMC9731513; doi:10.3389/fpls.2022.1031679)
Supplement: Supplementary file 11 [file Table_11.docx]

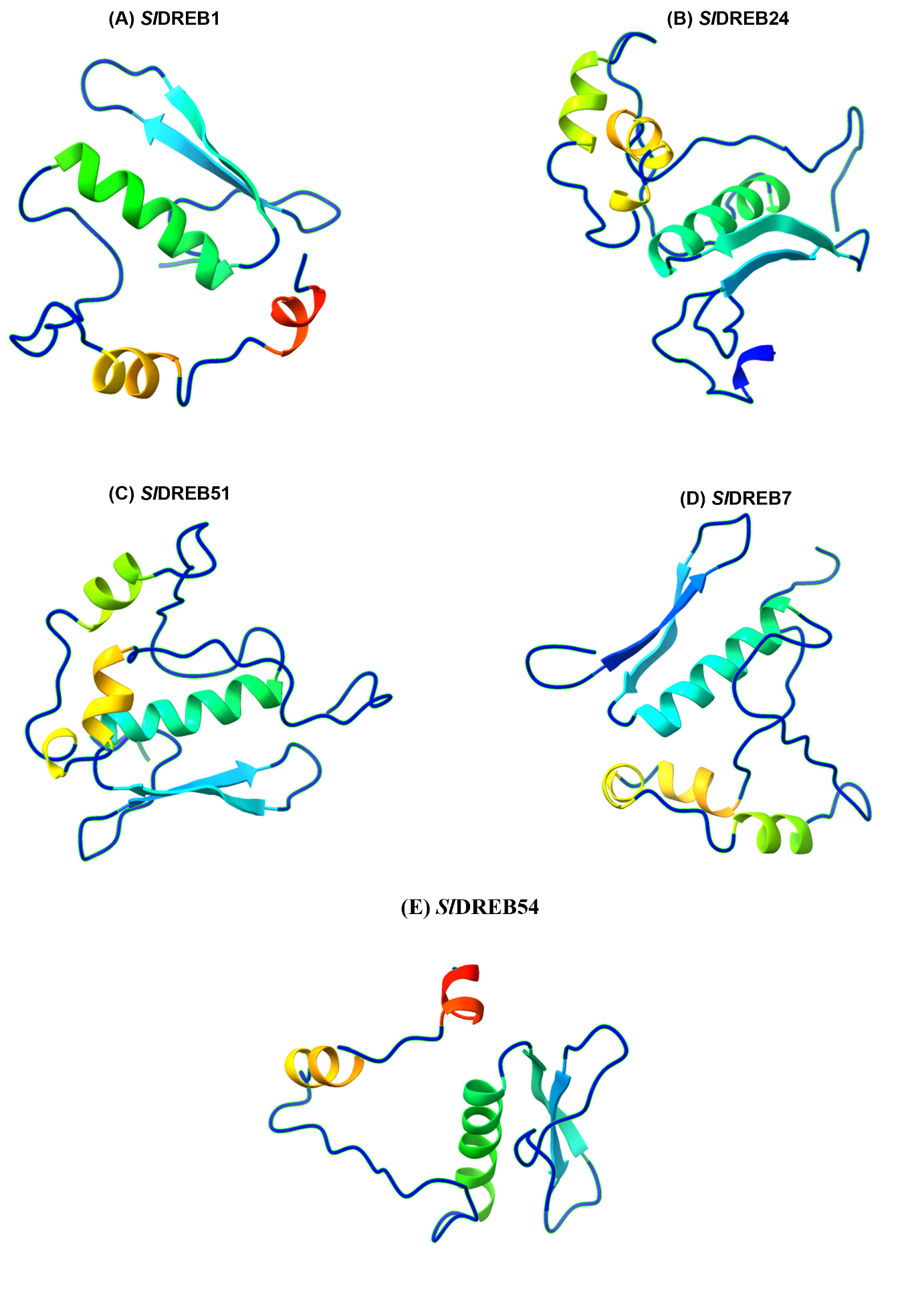


**Supplementary Figure**: Tertiary structures of five representatives of SlDREB proteins. The figure depicted the alpha helices and beta turns in each stable protein structure after the refinement from Galaxy refine server.
