# Supplementary material for: Genome-wide identification, comprehensive characterization of transcription factors, cis-regulatory elements, protein homology, and protein interaction network of DREB gene family in Solanum lycopersicum
Source: Front Plant Sci. 2022 Nov 24;13:1031679. doi: 10.3389/fpls.2022.1031679 (PMC9731513; doi:10.3389/fpls.2022.1031679)
Supplement: Supplementary file 12 [file Table_12.docx]

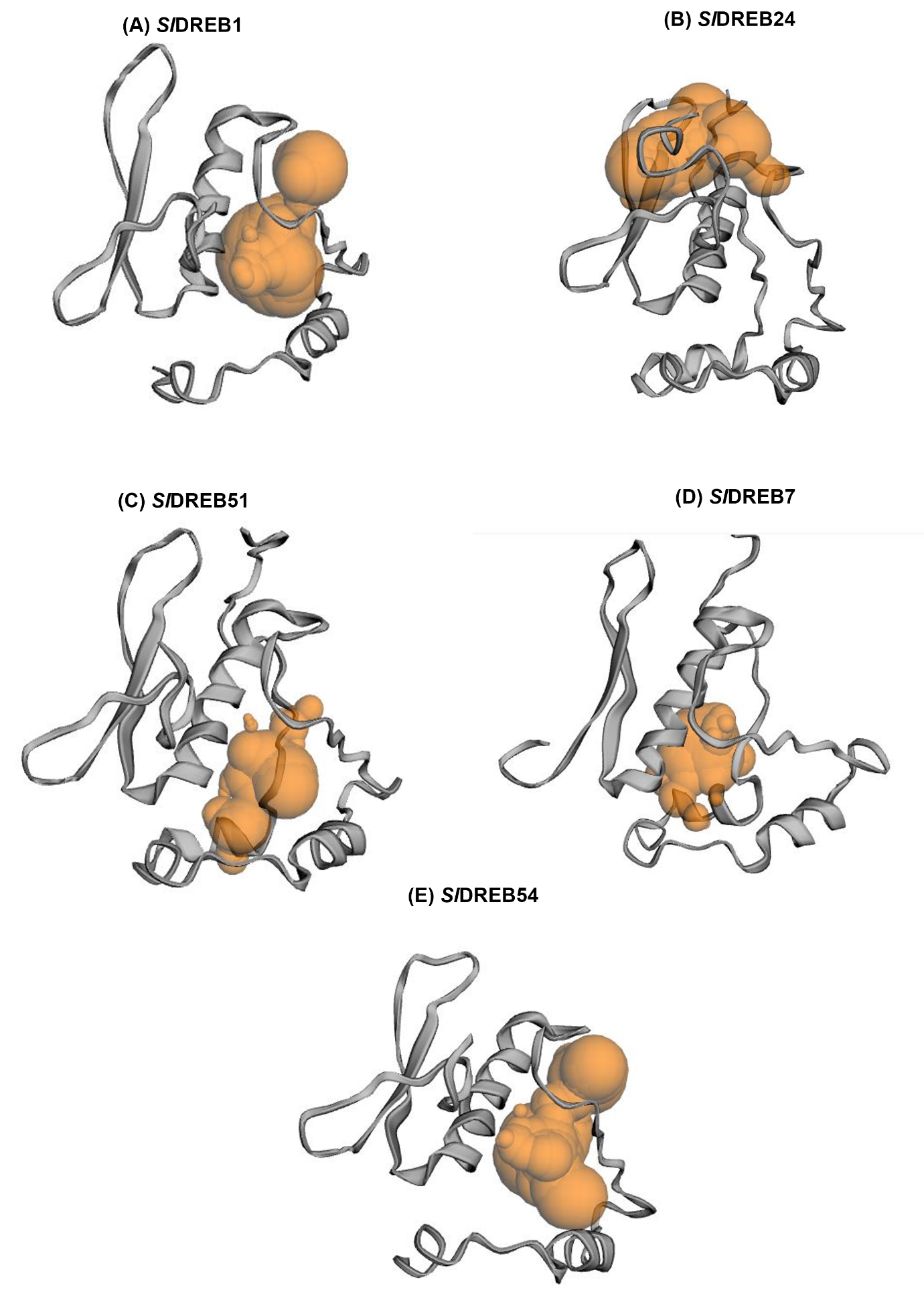


**Supplementary Figure: (A)** Prediction of active pockets in SlDREB protein structures. CASTp was employed that highlighted the active catalytic regions. These regions have the ability to interact with different interactors depending on the cellular conditions.


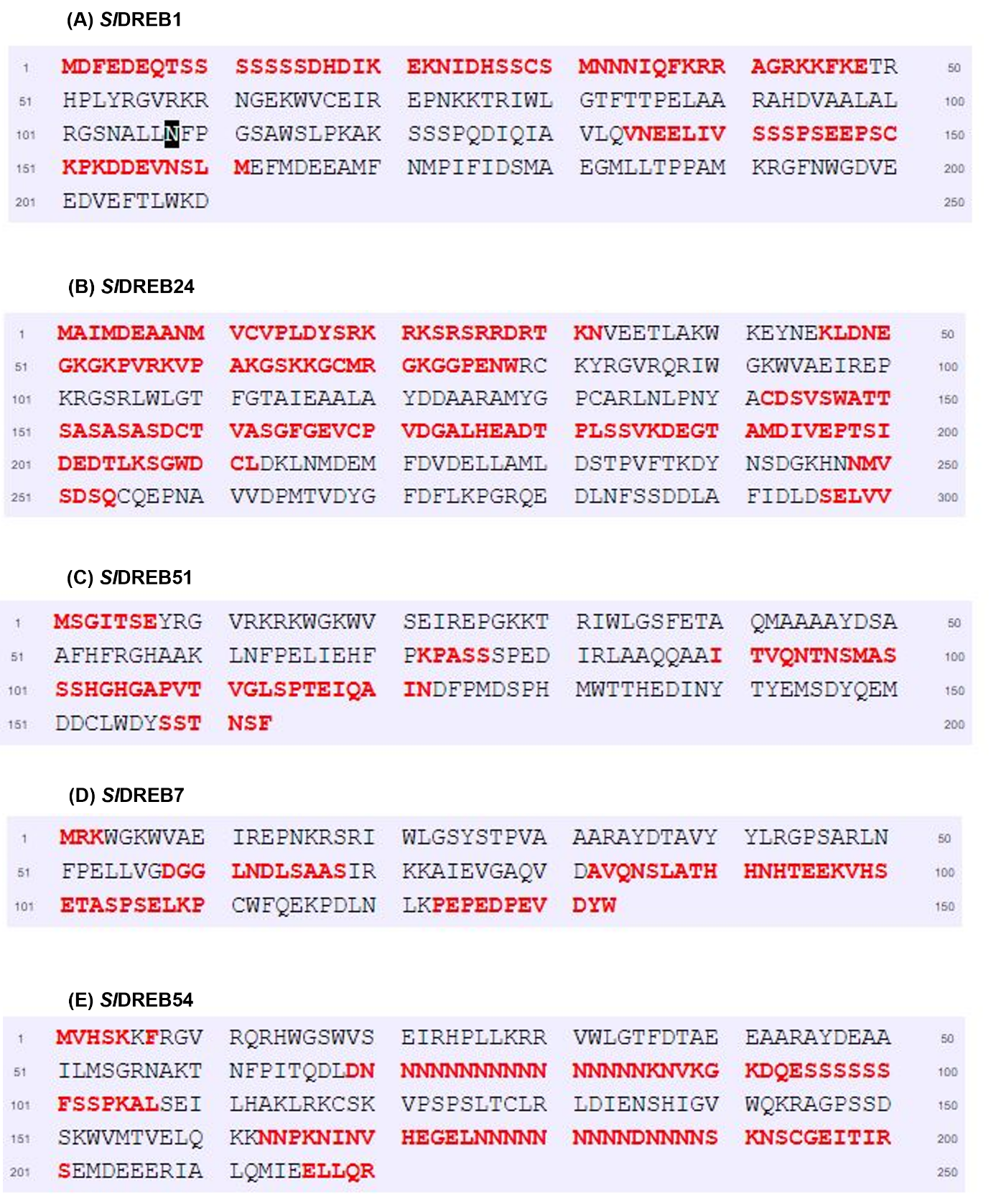


**Supplementary Figure: (B)** PrDOS server was utilized for the evaluation of disorder regions. Red regions depicted disorder regions in SlDREB proteins.
